# TRIM21 Promotes Tumor Growth and Gemcitabine Resistance in Pancreatic Cancer by Inhibiting EPHX1‐Mediated Arachidonic Acid Metabolism

**DOI:** 10.1002/advs.202413674

**Published:** 2024-12-30

**Authors:** Xiaona Fan, Yisheng Dai, Chuanfeng Mo, Hengzhen Li, Xindi Luan, Bojun Wang, Jiani Yang, Guangtao Jiao, XiaoLin Lu, Zhuo Chen, Yuanyu Liao, Ling Qu, Huike Yang, Changjie Lou, Chao Liu, Zhiwei Li

**Affiliations:** ^1^ Department of Gastrointestinal Medical Oncology Harbin Medical University Cancer Hospital Harbin 150081 P. R. China; ^2^ Key Laboratory of Tumor Immunology in Heilongjiang Harbin Medical University Cancer Hospital Harbin 150081 China; ^3^ Department of Orthopedic Surgery Harbin Medical University Cancer Hospital Harbin 150081 China; ^4^ Department of Anatomy Harbin Medical University Harbin 150081 China

**Keywords:** arachidonic acid metabolism, EPHX1, pancreatic cancer, TRIM21, ubiquitination

## Abstract

Pancreatic cancer (PC) progresses rapidly, and gemcitabine‐based chemotherapy has brought only limited efficacy. Identifying key drivers and therapeutic targets holds significant clinical value. In this study, through comprehensive analysis of multiple PC databases, this work identifies TRIM21 as a promising driver mediator. This work further performs loss‐ and gain‐of‐function assays for TRIM21, revealing that TRIM21 knockout inhibits tumor proliferation and gemcitabine resistance both in vitro and vivo. Lipidomics reveal that silencing TRIM21 reduce the arachidonic acid production, and inhibit ferroptosis. Mechanically, through proteomics, ubiquitomics, and liquid chromatography‐tandem mass spectrometry analysis, the key metabolic enzyme of arachidonic acid ‐EPHX1 is identified as a downstream substrate of TRIM21. TRIM21 interacts with EPHX1 through its SPRY domain and promotes ubiquitin‐mediated degradation of EPHX1 via K33‐ and K48‐linked ubiquitination at the K105 site. Given the targeting potential, this work screens Bezafibrate to block the interaction between TRIM21 and EPHX1 and validates its sensitizing effect. In summary, TRIM21 promotes tumour growth and gemcitabine resistance in PC by inhibiting EPHX1‐mediated arachidonic acid metabolism. This provides a novel and promising target for clinical treatment of PC.

## Graphical abstract

This study integrates multi‐omics to reveal TRIM21's pivotal role in pancreatic cancer development and resistance to gemcitabine. TRIM21 promotes ubiquitin‐mediated degradation of EPHX1 at the K105 site, which in turn inhibits arachidonic acid metabolism. Bezafibrate sensitizes gemcitabine sensitivity by inhibiting the TRIM21‐EPHX1 interaction and is a promising combination treatment strategy.

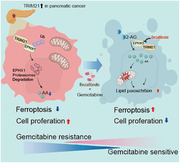

## 1 Introduction

Pancreatic cancer (PC) is among the most lethal malignancies, with a 5‐year survival rate of ≈10% and a mortality‐to‐incidence ratio of 94%.^[^ ^1^, ^2^, ^3^, ^4^ ^]^ Gemcitabine‐based therapies remain the preferred treatment for advanced pancreatic cancer. However, the clinical response rate is only ≈20%, with overall survival ranging from 6.7 to 10.1 months.^[^ ^5^, ^6^, ^7^ ^]^ The major driver genes of PC include KRAS (90%), TP53 (71%), CDKN2A (24%), and SMAD4 (17%).^[^ ^8^, ^9^ ^]^ Although these are well‐established driver genes in PC, their therapeutic potential as clinical drug targets remains limited.^[^ ^9^, ^10^ ^]^Therefore, further elucidation of the key events driving the malignant progression and gemcitabine resistance in PC is of great clinical value.

TRIM21, a RING‐domain E3 ubiquitin ligase, has been demonstrated to exert diverse biological processes such as tumorigenesis, progression, and therapy resistance.^[^ ^11^, ^12^, ^13^, ^14^ ^]^ TRIM21 accelerates liver cancer development by inhibiting p62 ubiquitination, thereby reducing the activity of the Keap1‐Nrf2 antioxidant pathway.^[^ ^11^ ^]^ TRIM21 targets TIF1γ for degradation and promotes the subcellular translocation of active β‐catenin, thereby driving glioblastoma progression.^[^ ^12^ ^]^ Moreover, TRIM21 also affects the resistance of cancer therapies. TRIM21 promotes resistance to radiotherapy though degradation of VDAC2 suppressing type I interferon responses.^[^ ^14^ ^]^ TRIM21 inhibits glioma cell senescence, promotes malignant phenotypes, and enhances temozolomide treatment resistance.^[^ ^15^ ^]^ In gastrointestinal stromal tumors, TRIM21 ubiquitinates ACSL4 leading to imatinib resistance.^[^ ^13^ ^]^ Nonetheless, the biological roles and molecular mechanisms of TRIM21 in PC has been limitedly reported.

Microsomal epoxide hydrolase 1 (EPHX1), as a conserved biotransformation enzyme, performs detoxification functions. EPHX1 regulates lipid metabolism pathways by catalyzing the decomposition of 2‐arachidonoylglycerol (2‐AG) into free arachidonic acid (AA) and glycerol.^[^ ^16^ ^]^ Typically, 2‐AG levels are elevated in epithelial tissue and contribute to tumorigenesis.^[^ ^17^, ^18^ ^]^ Impaired enzyme activity caused by the EPHX1 Tyr113His polymorphism is an increased risk element of various tumors.^[^ ^19^, ^20^ ^]^ Meanwhile, AA‐mediated lipid peroxidation induces ferroptosis in tumor cells and promotes the efficacy of anti‐tumor therapy.^[^ ^21^ ^]^ Therefore, maintaining the activity of EPHX1 is crucial for inhibiting tumor progression and enhancing anti‐tumor efficacy.

In current study, we identified TRIM21 as a potential prognostic marker and key gene in gemcitabine resistance in PC. Lipidomics, proteomics, ubiquitomics, and liquid chromatography‐tandem mass spectrometry(LC–MS/MS) analysis precisely defined TRIM21 downstream targets EPHX1. And EPHX1 mediates ferroptosis induced by AA to inhibit PC proliferation. Furthermore, docking small molecule drugs using their structure data file with the TRIM21 binding site identified Bezafibrate as a potential inhibitor of the TRIM21‐EPHX1 interaction. In summary, TRIM21 is a promising target for sensitizing of gemcitabine therapy in PC, providing a novel strategy for the treatment of this malignancy.

## 2 Results

### 2.1 TRIM21 is Upregulated in Pancreatic Cancer and Associated with Poor Response to Gemcitabine Treatment

**Figure 1:**
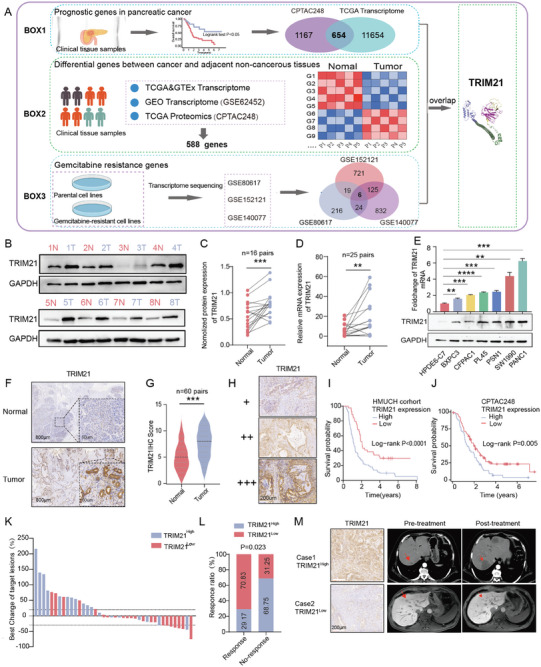
TRIM21 is upregulated in pancreatic cancer (PC) and is associated with poor response to gemcitabine treatment. A) Schematic diagram illustrating the process of identifying TRIM21 as a prognostic gene in PC and a gene associated with gemcitabine resistance. B,C) Western blot analysis showing TRIM21 protein level in fresh PC and adjacent non‐cancerous tissues. D) TRIM21 mRNA expression in fresh PC and adjacent non‐cancerous tissues (n = 25) using RT‐qPCR analysis. E) mRNA and Western blot analysis of TRIM21 expression in normal pancreatic ductal epithelial cell line HPDE6‐C7 and six PC cell lines. F) Immunohistochemistry (IHC) analysis confirming that TRIM21 expression were higher in tumor tissues compared to adjacent non‐cancerous tissues. Scale bars, 800 µm (exterior), 60 µm (interior). G) Statistical analysis of TRIM21 IHC staining intensity between tumor and adjacent tissues in the Harbin Medical University Cancer Hospital (HMUCH) cohort (n = 60). H) IHC staining intensity of TRIM21 was scored as 1 (+, weak), 2 (++, moderate), or 3 (+++, strong). I) Kaplan‐Meier survival analysis of PC patients in the HMUCH cohort (n = 116) correlating TRIM21 expression with survival probability. The log‐rank test: *p* < 0.0001. J) Kaplan‐Meier survival analysis in the CPTAC248 cohort correlating TRIM21 expression with survival probability. Statistical analysis was performed using the log‐rank test: *p* = 0.005. K) Correlation between TRIM21 expression and the therapeutic efficacy of gemcitabine in PC patients (n = 40). L) Statistical analysis of the correlation between TRIM21 expression and the therapeutic efficacy of gemcitabine in PC patients (n = 40) using the chi‐square test: *p* = 0.023. M. TRIM21 expression(left) in representative patients responding or not responding to gemcitabine and imaging changes before and after treatment (right). Statistical significance in the figures is indicated as follows: ns>0.05; ** for 0.001 ≤ *p* < 0.01; *** for 0.0001 ≤ *p* < 0.001; **** for *p* < 0.0001.

Here, using public proteomics data from CPTAC248 and transcriptomics data from the TCGA database, we identified 654 prognostic genes associated with pancreatic cancer. (**Figure** **1**A. Box 1). Subsequently, we conducted an analysis of transcriptomic data, incorporating TCGA, GTEx datasets, and the GEO database (GSE62452), as well as proteomic data (CPTAC248), resulting in the identification of 588 differentially expressed genes between pancreatic cancer and adjacent non‐cancerous tissues (all *p* < 0.05, Figure 1A. Box 2, Figure S1A, Supporting Information). To identify gemcitabine resistance‐related genes, we screened multiple gemcitabine‐resistant cell lines from GEO (GSE80617, GSE152121, GSE140077), resulting in the identification of 6 differentially expressed genes in resistant cell lines (all *p* < 0.001, Figure 1A, Box3). Finally, we identified TRIM21 is a pivotal gene linked to pancreatic cancer prognosis and gemcitabine resistance in pancreatic cancer. (Figure 1A, Figure S1B, Supporting Information). Western blot analysis of 16 paired fresh PC and adjacent non‐cancerous tissues demonstrated an increase in TRIM21 expression in the cancerous tissues (Figure 1B,C, Figure S1C, Supporting Information). RNA analysis showed similar results (Figure 1D). Compared to the normal pancreatic ductal epithelial cell line HPDE6‐C7, TRIM21 mRNA and protein levels were substantially elevated in pancreatic cancer cell lines (Figure 1E). We performed Immunohistochemistry (IHC) analysis on adjacent non‐cancerous and cancerous tissues (n = 60), serous cystadenomas (n = 33) and solid pseudopapillary neoplasms (SPN, n = 26), and the expression of TRIM21 in PC tumor tissues is higher than that in the other three types of tissues (Figure 1F,G, Figure S1D, Supporting Information). Based on TRIM21 expression, patients were classified into high and low expression groups, with elevated TRIM21 being associated with poor prognosis in PC(n = 116) (Figure 1H,I). Similarly, TRIM21 expression shows a correlation with overall survival in patients, as observed in the CPTAC248 protein database and the TCGA database (Figure 1J, Figure S1E, Supporting Information). Additionally, the expression level of TRIM21 increases with the clinical staging and pathological progression of pancreatic cancer (Figure S1F,G, Supporting Information). We also evaluated the expression of TRIM21 in patients received gemcitabine‐based agents as first‐line treatment after recurrence and metastasis following radical resection of PC (n = 40). The results revealed higher TRIM21 expression was associated with poorer therapeutic efficacy of gemcitabine in Harbin Medical University Cancer Hospital (HMUCH) cohort (gemcitabine response:17 versus 7; gemcitabine no‐response: 5 versus 11; *p* = 0.023) (Figure 1K,L). Representative TRIM21 expression level and imaging changes in the treatment cohort are shown in Figure 1M. The clinicopathological information of the PC patients was presented in Tables S1–S3, Supporting Information.

### 2.2 TRIM21 Promotes the Proliferation of Pancreatic Cancer in Vivo and Vitro

**Figure 2:**
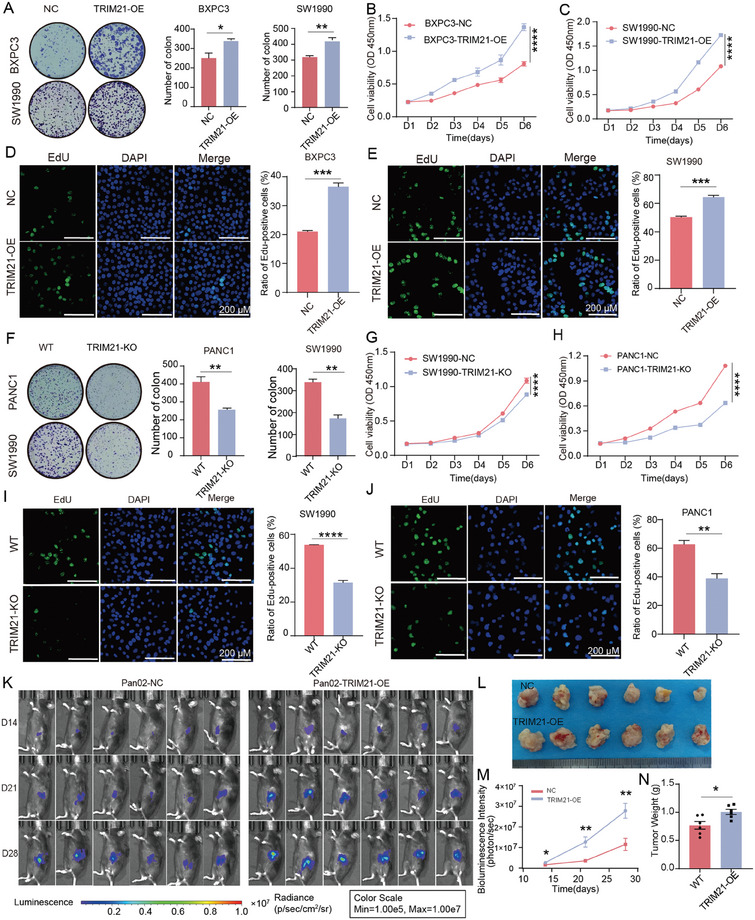
TRIM21 promotes the proliferation of pancreatic cancer in vivo and vitro. A–E) Effects of TRIM21 overexpression on the proliferation ability of the BXPC3 and SW1990 cell lines was measured using colony formation (A), CCK8 (B, C), and EdU (D, E). F–J) Effects of TRIM21 knockout on the proliferation ability of the SW1990 and PANC1 cell line was measured using colony formation (F), CCK8 (G, H), and EdU (I, J). K) Pan02 cells infected with pLV‐TRIM21‐luc virus or pLV‐control‐luc virus were used to establish C57BL/6 pancreatic cancer orthotopic mouse model(n = 6). Orthotopic mouses were performed in vivo imaging of mice on days 14, 21, and 28. L) Images of Orthotopic xenograft tumor in Pan02‐NC and Pan02‐TRIM21‐OE. M) Quantification of fluorescence values from in vivo imaging of the orthotopic mouse model(n = 6). N) Weight analysis of the two groups with orthotopic xenograft tumors(n = 6). Statistical analysis was performed using a two‐tailed unpaired *t*‐test in (A, D, E, F, I, J, M, N) and statistical analysis was performed using Two‐Way ANOVA in (B, C, G, H) Data are presented as mean ± SEM. Statistical significance in the figures is indicated as follows: ns > 0.05; * for *p* < 0.05; ** for 0.001 ≤ *p* < 0.01; and *** for 0.0001 ≤ *p* < 0.001, **** for *p* < 0.0001.

To investigate the role of TRIM21 in pancreatic cancer cells, we established TRIM21‐overexpressing (TRIM21‐OE) cell line models for BXPC3, SW1990, and Pan02, as well as TRIM21‐knockout (TRIM21‐KO) cell line models for SW1990 and PANC1 (Figure S2A,B, Supporting Information). Subsequently, cell proliferation was assessed using the CCK8, colony formation and EdU staining assay. The results indicated that overexpression of TRIM21 enhanced proliferation in SW1990 and BXPC3 cells (*P* < 0.0001) (**Figure** **2**B,C). The colony formation assay showed an increase in the number of colonies in BXPC3‐OE and SW1990‐OE (all *p* < 0.05) (Figure 2A). EdU staining assay confirmed that the TRIM21‐OE BXPC3 and SW1990 cells exhibited higher DNA replication activity (all *p* < 0.001) (Figure 2D,E). Conversely, functional assays demonstrated that TRIM21 deletion in SW1990 and PANC1 cells reduced cell proliferation (all *p* < 0.0001) (Figure 2G,H), colony formation capacity (*P* < 0.01) (Figure 2F), and DNA replication activity (all *p* < 0.01) (Figure 2I,J). However, transwell and wound healing assays revealed no significant impact of TRIM21‐OE and TRIM21‐KO on pancreatic cancer cell migration (Figure S2C–F, Supporting Information). In a C57BL/6 pancreatic cancer orthotopic mouse model, Pan02 cells infected with pLV‐TRIM21‐luc virus exhibited stronger bioluminescence intensity compared to pLV‐control‐luc cells (Figure 2K,M). And we indicated the tumor volume in the TRIM21‐OE group was larger than that in the control group (Figure 2L,N). These findings further confirm that TRIM21 functions as an oncogene, promoting PC cell proliferation both in vivo and in vitro.

### 2.3 TRIM21 Enhances Gemcitabine Resistance in Pancreatic Cancer

**Figure 3:**
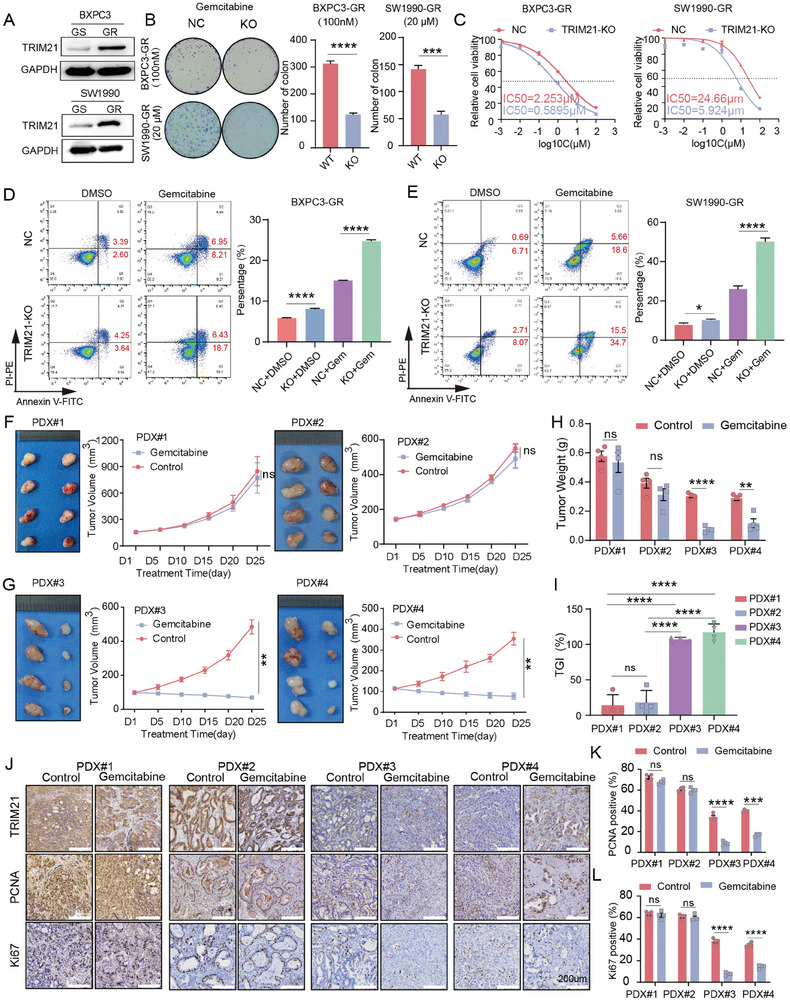
TRIM21 enhances gemcitabine resistance in pancreatic cancer. A) Western blot was performed to assess the expression of TRIM21 in the gemcitabine‐resistant or gemcitabine‐sensitive cell lines (BXPC3‐GR/GS and SW1990‐GR/GS). B) Colony formation assays were conducted following gemcitabine treatment in NC and TRIM21‐knockout BXPC3‐GR and SW1990‐GR cell lines. C) The changes in IC50 values were determined after TRIM21 knockout in BXPC3‐GR and SW1990‐GR cells. D,E) Annexin‐V/PI analysis was performed on NC and TRIM21‐knockout BXPC3‐GR (D) and SW1990‐GR (E) cell lines following gemcitabine treatment. F,G) In PDX models, tumor volume and growth curves were monitored in PDX#1, PDX#2 (F), PDX#3, and PDX#4 (G) following treatment with gemcitabine (50 mg kg^−1^ weekly) or an equal volume of saline (Control group) (n = 4). H) Tumor weights were measured and statistically analyzed between the gemcitabine and Control groups in PDX#1, PDX#2, PDX#3, and PDX#4 (n = 4). I) Tumor growth inhibition index (TGI) was calculated for PDX#1, PDX#2, PDX#3, and PDX#4 following gemcitabine treatment (n = 4), Data are presented as mean ± SD. J) Representative immunohistochemistry staining of TRIM21, PCNA, and KI67 from tumors of PDX models. Scale bars, 200 µm. K, L) Quantitative analysis of PCNA(K) and KI67(L). Statistical analysis was performed using a two‐tailed unpaired *t*‐test in (B, D, E, H, I, K, L), and statistical analysis was performed using Two‐Way ANOVA in (F, G), Data are presented as mean ± SEM. Statistical significance in the figures is indicated as follows: ns > 0.05; * for *p* < 0.05; ** for 0.001 ≤ *p* < 0.01; *** for 0.0001 ≤ *p* < 0.001; and **** for *p* < 0.0001.

To explore the function of TRIM21 in gemcitabine resistance in PC, we established gemcitabine‐resistant pancreatic cancer cell lines BXPC3‐GR and SW1990‐GR (Figure S3A, Supporting Information). We observed an increase in TRIM21 mRNA and protein levels in the resistant cell lines (**Figure** **3**A; Figure S3B, Supporting Information). Next, we generated TRIM21 knockout cell lines from BXPC3‐GR and SW1990‐GR (Figure S3C, Supporting Information). Colony formation assays TRIM21 silencing enhanced the inhibitory effect of gemcitabine on cell proliferation (Figure 3B). The CCK‐8 assay demonstrated that TRIM21 silencing attenuated the resistance of pancreatic cancer cells to gemcitabine (Figure 3C). Annexin‐V/PI analysis revealed that TRIM21 knockout combined with gemcitabine treatment increased the proportion of apoptotic and necrotic cells compared to gemcitabine treatment in NC (Figure 3D,E). We also proved this changes in apoptosis‐related protein markers (Figure S3D, Supporting Information). Similarly, we found that overexpression of TRIM21 in the parental BXPC3 and SW1990 cells reduced gemcitabine‐induced apoptosis compared with the NC (Figure S3E,F, Supporting Information). First, we evaluated the basal TRIM21 expression in the patients used for constructing PDX models (Figure S3G, Supporting Information). Using pancreatic cancer PDX model (n = 4), we demonstrated PDX#1 and PDX#2 (TRIM21^high^, Figure 3J) exhibited resistance to gemcitabine treatment (Figure 3F), while PDX#3 and PDX#4 (TRIM21^low^, Figure 3J) were sensitive to gemcitabine (Figure 3G). PDX#3 and PDX#4 showed more pronounced tumor volume reduction and tumor growth inhibition index (TGI), compared to PDX#1 and PDX#2 (Figure 3H,I). The expression of KI67 and PCNA in the tumor tissues of PDX#1 and PDX#2 showed no differences after gemcitabine treatment, while in PDX#3 and PDX#4, their expression was significantly reduced post‐treatment (Figure 3J–L). Hematoxylin and eosin (HE) staining was performed on the tissues of PDX mice. Among them, PDX#3 and PDX#4 showed obvious changes of tumor tissue structure regression after treatment (Figure S3H, Supporting Information). Clinical and pathological information of patients for PDX model in Table S4, Supporting Information.

### 2.4 TRIM21 Promotes Tumors Through Interacting with and Enhancing Degradation EPHX1

**Figure 4:**
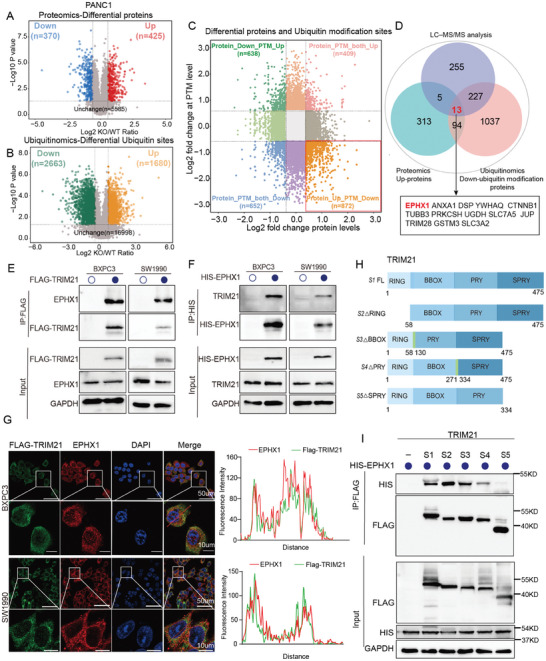
TRIM21 promotes tumors through interacting with and enhancing degradation EPHX1. A,B) Volcano plots display the differentially expressed proteins (A) and ubiquitin modification sites (B) between PANC1‐WT and PANC1‐TRIM21‐knockout cell (|Fold change| > 1.5, *p* < 0.05). C) A nine‐quadrant plot illustrates the integrated analysis of proteomics and ubiquitinomics, with each quadrant showing the differentially expressed proteins and ubiquitin modification sites with a threshold of |Fold change| > 1.5 and *p* < 0.05. D) The Venn diagram represents the intersection of proteins that were upregulated in PANC1‐TRIM21‐KO and protein with decreased ubiquitination sites, following LC–MS/MS analysis of PANC1 overexpressing TRIM21. E) Immunoprecipitation was performed in BXPC3 and SW1990 cells after overexpressing FLAG‐TRIM21, with WT used as a negative control. Immunoprecipitation was performed using FLAG‐magnetic beads. F) Immunoprecipitation was performed in BXPC3 and SW1990 cells after overexpressing HIS‐EPHX1, with WT used as a negative control. Immunoprecipitation was performed using HIS‐magnetic beads. G) Laser confocal microscopy images show the co‐localization of FLAG‐TRIM21 and EPHX1 in BXPC3 and SW1990 cells. The scale bars represent 50 (upper) and 10 µm (lower). H) The strategy for constructing truncated TRIM21 plasmids. I) HEK293T cells were transfected with HIS‐EPHX1 and indicated full‐length or truncated mutants of FLAG‐TRIM21. Co‐immunoprecipitation was used to detect the interaction between EPHX1 and the truncated regions of TRIM21.

Next, we performed proteomic and ubiquitomic sequencing on PANC1 and PANC1‐TRIM21‐KO cells. In the proteomic analysis, we identified 425 upregulated and 370 downregulated proteins in the TRIM21‐KO group compared to the PANC1‐WT group (**Figure** **4**A). In the ubiquitomic analysis, we detected 1680 (38.68%) upregulated ubiquitination sites and 2663 (61.32%) downregulated ubiquitination sites in the TRIM21‐KO group (Figure 4B). Based on these results, we revealed that 107 proteins with increased quantification had a total of 872 sites with decreased ubiquitination in TRIM21‐KO group (Figure 4C,D, Figure S4A, Supporting Information). KEGG enrichment analysis of the differentially expressed proteins and ubiquitination sites revealed associations with pathways such as the proteasome, ubiquitin‐mediated proteolysis, fatty acid metabolism, and ferroptosis (Figure S4B, Supporting Information). Furthermore, we performed LC–MS/MS analysis on PANC1 overexpressing TRIM21 to identify proteins potentially interacting directly with TRIM21. Details of the LC–MS/MS analysis are provided in Supp‐0002‐SuppMat 1. By intersecting proteomic, ubiquitomic, and LC–MS/MS data, we identified 13 proteins (Figure 4D). Gene Set Enrichment Analysis (GSEA) indicated that the differentially expressed proteins are positively correlated with the lipid metabolism signature (*P* < 0.001) (Figure S4C, Supporting Information), with EPHX1 being one of the key genes in the lipid metabolism pathway.^[^ ^16^ ^]^ We conducted Co‐immunoprecipitation (Co‐IP) experiments with BXPC3 and SW1990 cells overexpressing FLAG‐TRIM21, which demonstrated an interaction between TRIM21 and EPHX1 (Figure 4E). Similarly, Co‐IP experiments with BXPC3 and SW1990 cells overexpressing HIS‐EPHX1 yielded consistent results (Figure 4F). Furthermore, endogenous interactions between TRIM21 and EPHX1 were confirmed in the BXPC3 and SW1990 (Figure S4D,E, Supporting Information). To further clarify the interaction between EPHX1 and TRIM21, we performed immunofluorescence experiments, which revealed colocalization of the two proteins (Figure 4G). We also constructed a series of FLAG‐TRIM21 deletion mutants (Figure 4H). Co‐transfection of these deletion mutants with HIS‐EPHX1 into HEK293T cells, followed by Co‐IP, showed that the SPRY domain of TRIM21 is critical for binding to EPHX1, as the SPRY domain deletion mutant lost the ability to interact with EPHX1 (Figure 4I). The AlphaFold2 approach demonstrates the modeled complex structure of TRIM21 and EPHX1, as well as their interactions (Figure S5C, Supporting Information).

### 2.5 TRIM21 Degrades EPHX1 Via K33‐ and K48‐Linked Poly‐Ubiquitination at Lysine 105

**Figure 5:**
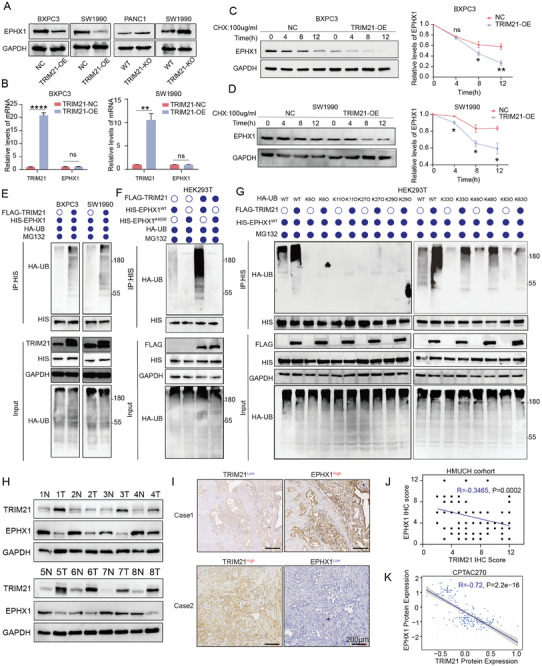
TRIM21 via promoting K33‐and K48‐linked poly‐ubiquitinates EPHX1 at lysine 105. A) Detecting EPHX1 protein expression following TRIM21 overexpression and knockout. B) RT‐qPCR was used to measure EPHX1 mRNA expression after TRIM21 overexpression. C,D) Western blot analysis was conducted on BXPC3 and SW1990 cells overexpressing TRIM21, which were treated with cycloheximide (CHX), to assess the remaining EPHX1 protein levels at specific time points. E) Co‐IP followed by western blotting was used to detect the polyubiquitination levels of EPHX1 in BXPC3 (left) and SW1990 (right) cells transfected with HIS‐EPHX1, FLAG‐TRIM21, and HA‐UB. F) Co‐IP followed by western blotting was used to detect the polyubiquitination levels of EPHX1 in HEK293T cells were transfected with HA‐UB and either HIS‐EPHX1 or HIS‐EPHX1^K105R^ (lysine 105 to arginine 105 mutant), with or without FLAG‐TRIM21. G) HEK293T cells were transfected with HIS‐EPHX1, FLAG‐TRIM21, and HA‐ubiquitin mutants at different sites, followed by Co‐IP and Western blot analysis. H) Western blot analysis was performed to detect TRIM21 and EPHX1 protein levels in fresh pancreatic cancer tissues and adjacent non‐tumor tissues(n = 8). I) Representative immunohistochemistry images showing the protein expression of EPHX1 and TRIM21. Scale bars, 200 µm. J) Immunohistochemistry analysis was used to evaluate the correlation between EPHX1 and TRIM21 protein expression (n = 112, Pearson correlation test). K) Pearson correlation analysis of EPHX1 and TRIM21 protein levels in a public proteomics dataset (CPTAC270). All Co‐IP experiments for ubiquitination detection were conducted 48 h post‐transfection, followed by a 4‐h treatment with MG132. Data are presented as mean ± SEM. Statistical analysis used two‐tailed unpaired *t*‐tests for (B, C, D) significance in the figures is indicated as follows: ns > 0.05; * for *p* < 0.05; ** for 0.001 ≤ *p* < 0.01; *** for 0.0001 ≤ *p* < 0.001; and **** for *p* < 0.0001.

Next, we further confirmed that TRIM21 overexpression resulted in a reduction in EPHX1 protein levels, whereas silencing TRIM21 caused an increase in EPHX1 protein levels (**Figure** **5**A). However, EPHX1 mRNA levels did not show significant changes (Figure 5B). After treatment with proteasome inhibitor MG132, the expression of EPHX1 increased (Figure S5A,B, Supporting Information). Upon cycloheximide (CHX) treatment, TRIM21 overexpression led to a reduction in EPHX1 protein levels compared to the NC group (Figure 5C,D). Co‐ IP experiments in the BXPC3, SW1990, and HEK293T cell lines all indicated that TRIM21 facilitates the ubiquitination of EPHX1 (Figure 5E, Figure S5F, Supporting Information). Additionally, increasing TRIM21 expression gradually resulted in a corresponding increase in EPHX1 ubiquitination levels (Figure S5G, Supporting Information). Ubiquitomics revealed multiple ubiquitination sites on EPHX1, and we propose that TRIM21 mediates ubiquitination at lysine 105(K105) of EPHX1 (Figure S5D, Supporting Information). We constructed a plasmid with the HIS‐EPHX1^K105R^ (lysine 105 to arginine 105 mutant). Co‐ IP experiments demonstrated that ubiquitination levels of HIS‐EPHX1^K105R^ were significantly reduced compared to HIS‐EPHX1^WT^ (Figure 5F). However, Co‐IP experiments showed that HIS‐EPHX1^K105R^ does not affect the interaction between TRIM21 and EPHX1 (Figure S5E, Supporting Information). To investigate the modification of EPHX1 by lysines on the ubiquitin chain, we constructed HA‐ubiquitin mutants retaining only single lysine residues, including K6O, K11O, K27O, K29O, K33O, K48O, and K63O. Co‐expression of FLAG‐TRIM21, HIS‐EPHX1, and wild‐type or mutant HA‐ubiquitin, followed by Co‐IP experiments, revealed that TRIM21 enhances polyubiquitination of EPHX1 protein at the K33‐linked and K48‐linked chains (Figure 5G). Finally, we examined the protein expression of TRIM21 and EPHX1 in fresh PC and the adjacent non‐tumorous tissues (n = 8) and in paraffin‐embedded tissue sections (n = 112). We observed a negative correlation between the protein level of TRIM21 and EPHX1(Figure 5H–J). Proteomics data from public databases (CPTAC270) also indicated a marked negative regulatory relationship between TRIM21 and EPHX1 (R = −0.72, *p* < 0.0001) (Figure 5K).

### 2.6 EPHX1 Mediates Ferroptosis Induced by AA to Inhibit PC Proliferation

**Figure 6:**
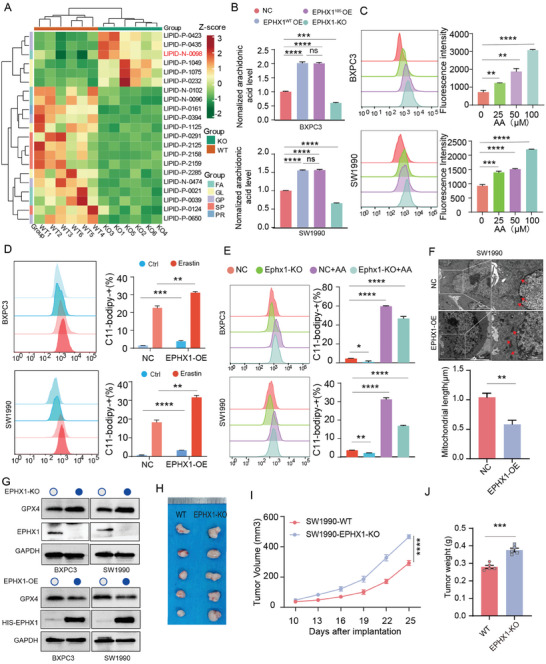
EPHX1 mediates ferroptosis induced by arachidonic acid (AA) to inhibit PC proliferation A) Heatmap illustrated the top five most altered class I lipid metabolites in the PANC1‐knockout (KO) group (n = 6, *p* < 0.05), with LIPID‐N‐0098 (arachidonic acid, AA) highlighted in red. B) ELISA was conducted to assess changes in AA after interfering with EPHX1 in BXPC3 and SW1990 cell lines. C) Flow cytometry measured changes in ROS fluorescence intensity after treating BXPC3 and SW1990 cell lines with different concentrations of AA for 72 h. D) Flow cytometry assessed the percentage of C11‐BODIPY‐+ (%) following treatment with Erastin. E) Flow cytometry compared the percentage of C11‐BODIPY+ (%), between the WT group and the EPHX1‐KO group, with or without AA (100 µM, 72 h). F) Transmission electron microscopy images of the EPHX1‐OE group exhibit mitochondrial morphological alterations characteristic of ferroptosis. G) Western blotting analyzed GPX4 expression levels in BXPC3 and SW1990 cell lines, after EPHX1 overexpression or knockout. H, I, J) Tumor size, tumor weight, and tumor growth curves were compared between the SW1990‐WT and SW1990‐EPHX1‐knockout (KO) groups in a subcutaneous xenograft BALB/c‐nude mice model. Data are presented as mean ± SEM. Statistical analysis used two‐tailed unpaired *t*‐tests for (B, C, D, E, F, J), and two‐way ANOVA for I) Statistical significance in the figures is indicated as follows: ns > 0.05; * for *p* < 0.05; ** for 0.001 ≤ *p* < 0.01; *** for 0.0001 ≤ *p* < 0.001; and **** for *p* < 0.0001.

To further elucidate the function of TRIM21 in lipid metabolism in PC, we conducted quantitative lipidomics analysis of lipid metabolites in PANC1‐WT and PANC1‐TRIM21‐KO cells. The results illustrated the level of AA was increased in the TRIM21‐KO group (**Figure** **6**A). See Sup‐0003‐SuppMat 2 for quantitative lipidomics analysis data. We hypothesized that TRIM21 exerts its biological function by affecting EPHX1 and thereby altering AA levels. We then generated BXPC3 and SW1990 cell lines with HIS‐EPHX1^WT^, HIS‐EPHX1^K105R^, and EPHX1‐KO constructs. Overexpression of HIS‐EPHX1^WT^ and HIS‐EPHX1^K105R^ resulted in an increase in AA levels, whereas AA levels decreased following EPHX1‐KO (Figure 6B). Initially, we observed that EPHX1 overexpression (EPHX1‐OE) reduced pancreatic cancer cell proliferation, while EPHX1 knockout (EPHX1‐KO) promoted proliferation, without affecting pancreatic cancer metastasis (Figure S6A–C, Supporting Information). We hypothesize that this biological effect results from EPHX1's influence on AA‐induced ferroptosis. To explore this, we exposed BXPC3 and SW1990 cell lines to different concentrations of AA for 72 h. We found that increased AA concentrations led to higher ROS production and decreased GPX4 expression (Figure 6C, Figure S6E, Supporting Information). Immunofluorescence also detected increased ROS production induced by AA (Figure S6D, Supporting Information). Subsequently, we observed that EPHX1‐OE increased lipid peroxidation compared to the NC group. Lipid peroxidation levels were notably elevated by the ferroptosis inducer Erastin (Figure 6D). After EPHX1 knockout (EPHX1‐KO), lipid peroxidation levels were reduced, but supplementation with AA restored the lipid peroxidation levels (Figure 6E). Transmission electron microscopy (TEM) was applied to visually assess the EPHX1‐OE group exhibited more pronounced mitochondrial shrinkage ferroptotic features (Figure 6F). We also measured GPX4 protein expression and found that EPHX1‐OE reduced GPX4 levels. AA supplementation mitigated the GPX4 increase induced by EPHX1 silencing (Figure 6G, Figure S6F, Supporting Information). The malondialdehyde (MDA) production in the EPHX1‐KO group cells was reduced, while the MDA levels notably increased after the supplementation of AA (Figure S6G, Supporting Information). Furthermore, we assessed the in vivo proliferation phenotype of EPHX1. The results indicated that the SW1990‐EPHX1‐KO group had a stronger proliferative capacity compared to the SW1990‐WT group (Figure 6H–J). Furthermore, the AA levels decreased in the EPHX1‐KO group of subcutaneous xenografts and the TRIM21‐OE group of orthotopic xenografts (Figure S6H,I, Supporting Information). In the PDX models, PDX#3 and PDX#4 (sensitive to gemcitabine) exhibited higher AA levels in tumor tissues compared to PDX#1 and PDX#2 (resistant to gemcitabine) (Figure S6J, Supporting Information).

### 2.7 Bezafibrate Sensitizes Gemcitabine by Preventing the Interaction Between TRIM21 and EPHX1

**Figure 7:**
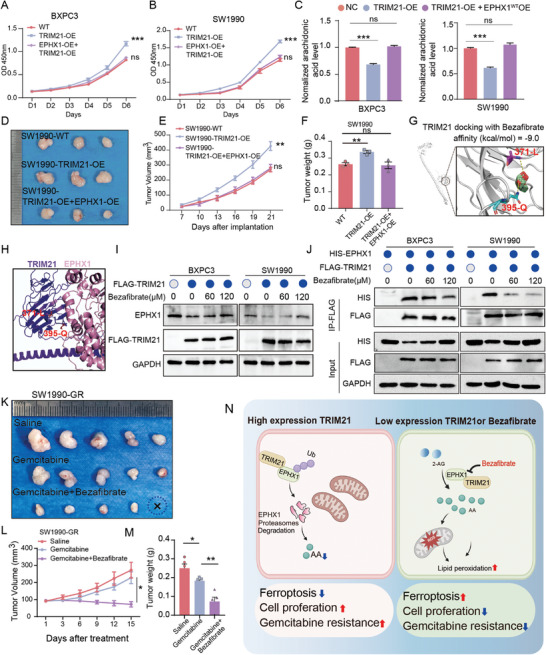
Bezafibrate sensitizes gemcitabine by preventing the interaction between TRIM21 and EPHX1. A, B) The proliferation of WT group, TRIM21‐OE group, and TRIM21 and EPHX1 co‐overexpression group in BXPC3 and SW1990. C) ELISA results show the AA levels in WT group, TRIM21‐OE group, and TRIM21 and EPHX1 co‐overexpression group in BXPC3 and SW1990. D–F) Tumor images, tumor volume, and tumor growth curves of subcutaneous xenograft experiments were assessed in SW1990 for the WT group, TRIM21‐OE, and TRIM21 and EPHX1 co‐overexpression groups. G) The molecular docking between Bezafibrate and the TRIM21‐SPRY domain was performed using AutoDock Vina software and visualized with PyMOL software. H) The AlphaFold2 approach revealed that the binding site of TRIM21 to Bezafibrate is a critical site for its interaction with EPHX1. I) Western blot analysis of EPHX1 protein levels in BXPC3 and SW1990 cells after 48 h of treatment with varying concentrations of Bezafibrate. J) Co‐immunoprecipitation (Co‐IP) were conducted after 48 h of treatment with different concentrations of Bezafibrate following the transfection of FLAG‐TRIM21 and HIS‐EPHX1 into BXPC3 and SW1990 cells. K, L) Subcutaneous xenograft experiments using SW1990‐GR cells were conducted, and tumor volume, growth curves, and tumor weight were assessed following treatments with saline, gemcitabine, and gemcitabine + Bezafibrate (n = 5). M) Tumor weights of SW1990‐GR subcutaneous xenografts after different treatment methods. **N**) The schematic illustrates that TRIM21 reduces the production of AA and inhibits ferroptosis by down‐regulating the expression of EPHX1, thereby promoting tumor growth and gemcitabine resistance. A, B, E, L) were analyzed using one‐way ANOVA followed by Tukey's multiple comparisons test, and C, F, M) were analyzed using two‐tailed unpaired *t*‐tests. Data are presented as mean ± SEM. Statistical significance in the figures is indicated as follows: ns > 0.05; * for *p* < 0.05; ** for 0.001 ≤ *p* < 0.01; *** for 0.0001 ≤ *p* < 0.001; and **** for *p* < 0.0001.

To further clarify whether EPHX1 can counteract the pro‐cancer effects of TRIM21, we co‐expressed HIS‐EPHX1 and FLAG‐TRIM21 in BXPC3 and SW1990 cell lines (Figure S7A, Supporting Information). CCK8 assays showed that simultaneous overexpression of EPHX1 and TRIM21 resulted in reduced cell growth compared to cells with TRIM21 overexpression alone (**Figure** **7**A,B). ELISA revealed that TRIM21 overexpression led to decreased AA production, while simultaneous overexpression of EPHX1 and TRIM21 restored AA levels to those observed in the NC group (Figure 7C). Additionally, TRIM21 overexpression led to a decrease in MDA levels, decrease of reduced glutathione (GSH) depletion, and a reduction in lipid peroxidation. However, after restoring EPHX1 expression, these levels showed almost no change (Figure S7B–D, Supporting Information). In vivo, tumors with TRIM21 overexpression exhibited larger volumes compared to both the NC group and the group with simultaneous overexpression of EPHX1 and TRIM21 (Figure 7D–F). Given the critical role of TRIM21 in pancreatic cancer, we conducted a small molecule drug screening using lipid modifying agents to target the binding domain between TRIM21 and EPHX1. Bezafibrate, a triglyceride‐lowering drug and a peroxisome proliferator‐activated receptor (PPAR)‐α agonist, may interact with TRIM21 at the 371‐L and 395‐Q sites (affinity (kcal mol^−1^) = −9.0) (Figure 7G). Table S5, Supporting Information showed lipid modifying agents of DrugBank database. AlphaFold2 protein docking of TRIM21 and EPHX1 indicates 371‐L and 395‐Q are critical binding sites for EPHX1 (Figure 7H). To further prove Bezafibrate function, we found that treatment with Bezafibrate rescues the reduction of EPHX1 induced by TRIM21 in BXPC3 and SW1990 cells (Figure 7I). Co‐IP experiments after Bezafibrate treatment in BXPC3 and SW1990 cells show a dose‐dependent decrease in the binding of FLAG‐TRIM21 to HIS‐EPHX1 (Figure 7J). Gemcitabine sensitivity studies using PANC1 and SW1990‐GR cell lines reveal the combination of Bezafibrate and gemcitabine is more effective than gemcitabine monotherapy (Figure S7E, Supporting Information). Finally, subcutaneous tumor implantation experiments with SW1990‐GR cells further confirm that the combination of Bezafibrate sensitizes gemcitabine in vivo (Figure 7K–M). Moreover, H&E staining of additional organs, including the heart, liver, spleen, lung and kidneys, revealed no significant damage across the different treatment groups (Figure S7F, Supporting Information).

## 3 Discussion

Pancreatic cancer often presents with no obvious symptoms in its early stages, and the majority of patients cannot be diagnosed at an early stage. Gemcitabine‐based regimens are a cornerstone in the management of pancreatic cancer at present. Therefore, it is essential to further investigate the mechanisms underlying pancreatic cancer progression and gemcitabine resistance. Our study identified TRIM21‐EPHX1 axis reduces AA production, protecting cells from ferroptosis and promoting cancer growth. Multi‐omics analysis revealed EPHX1, an enzyme involved in AA production, as a novel TRIM21 substrate. TRIM21 mediates EPHX1 degradation via ubiquitination at lysine 105 and facilitates the linkage of K33‐ and K48‐linked polyubiquitin chains. We found that Bezafibrate disrupts the TRIM21‐EPHX1 interaction sensitizing gemcitabine (Figure 7N).

As an E3 ubiquitin ligase, TRIM21 targets substrates for proteasomal degradation via K48‐linked polyubiquitination, while also utilizing K63‐linked polyubiquitination to maintain protein stability or other biological functions.^[^ ^12^, ^22^, ^23^, ^24^, ^25^, ^26^ ^]^ In this study, we revealed that in addition to K48‐polyubiquitination, TRIM21 can also promote the ubiquitination of EPHX1 through enhancing the linkage of K33‐linked chains. K33 ubiquitin chain modification is one of the atypical ubiquitination modifications with extremely low abundance in cells, and its function has not yet been fully elucidated. Research indicates that K33‐linked ubiquitination frequently co‐occurs with other ubiquitin chains, resulting in enhanced protein ubiquitination and facilitating protein instability and degradation.^[^ ^27^, ^28^ ^]^ K33‐linked ubiquitin chain modifications also can modulate the subcellular localization of proteins, thereby impacting their biological functions.^[^ ^29^ ^]^ Hence, the functions of K33‐linked chains will be further investigated in future research to explore their potential additional biological roles and implications.

Here, we identified that TRIM21 degrades EPHX1 through the proteasomal pathway using multiple omics approaches. In our study, lysine 105 of EPHX1 was pinpointed as the critical site for TRIM21‐induced ubiquitination. The K105 mutation reduced the ubiquitination level of EPHX1. However, the K105R mutation did not affect the binding between EPHX1 and TRIM21, nor did it impact the enzymatic activity of EPHX1. According to previous studies, oxidized arachidonic and adrenic phosphatidylethanolamines are crucial mediators of ferroptosis, and AA supplementation was proved to restore ferroptosis sensitivity in intestinal‐type gastric cancer.^[^ ^21^, ^30^ ^]^ Although studies have shown that low doses of AA can promote cell proliferation partially, the different outcomes in our study may be attributed to variations in the duration and concentration of AA treatment.^[^ ^31^ ^]^

In recent years, TRIM21 has been confirmed to play diverse roles in various tumors, due to its wide range of substrates.^[^ ^11^, ^12^ ^]^ TRIM21 could promote proliferation, migration, invasion, and stemness in cervical squamous cell carcinoma.^[^ ^32^ ^]^ Jun Gong et al. discovered that TRIM21 enhances the function of FSP1 by promoting K63‐linked ubiquitination, thereby preventing ferroptosis.^[^ ^33^ ^]^ Conversely, TRIM21promotes the ubiquitination and degradation of p53 mutant and subsequently inhibiting tumorigenesis.^[^ ^34^ ^]^ In colorectal and breast cancers, TRIM21 increases the degradation of SNAIL, thus decreasing epithelial‐mesenchymal transition and tumor metastasis.^[^ ^35^, ^36^ ^]^Given the dual roles of TRIM21, a targeted approach regulating the protein levels of its downstream substrates is likely to be more feasible and safer than broadly modulating TRIM21 expression. In this context, our study identifies Bezafibrate as a molecule that targets the interaction between TRIM21 and EPHX1. By preventing TRIM21‐mediated ubiquitination and subsequent degradation of EPHX1, Bezafibrate sensitizes pancreatic cancer cells to gemcitabine, offering a novel and promising therapeutic strategy. Besides, Bezafibrate combined gemcitabine treatment demonstrated controllable therapeutic toxicity in mouse experiments, and other lipid‐modifying agents such as simvastatin and candesartan are safe and exhibit manageable drug toxicity used in combination with gemcitabine for clinical study.^[^ ^37^, ^38^ ^]^

In summary, pancreatic cancer remains one of the most lethal malignancies. Despite the identification of numerous genetic mutations and oncogenic pathways, few have successfully translated into clinically actionable therapeutic targets. In this context, our study identified TRIM21 as a critical driver of pancreatic cancer progression and gemcitabine resistance. TRIM21 plays a pivotal role in tumor growth and chemoresistance by regulating EPHX1‐mediated arachidonic acid metabolism. More importantly, we demonstrated that inhibiting the interaction between TRIM21 and EPHX1 with Bezafibrate significantly sensitized pancreatic cancer cells to gemcitabine. In future research, we will further investigate the translational potential of Bezafibrate in both preclinical and clinical studies. These findings not only offer a novel perspective for improving the efficacy of chemotherapy in pancreatic cancer but also present a promising strategy to overcome drug resistance.

## 4 Experimental Section

### Cell Culture

Normal pancreatic ductal epithelial cell line (HPDE6‐C7), human pancreatic cancer cell lines (BXPC3, PANC1, SW1990, PL45, PSN1 and CFPAC1) and mouse cell lines (Pan02), and HEK239T cells were obtaineded from the Cell Bank of the Chinese Academy of Sciences. BXPC3 was maintained in Roswell Park Memorial Institute (RPMI) 1640 (Gibco, USA) SW1990, PL45, PSN1, PANC1, HPDE6‐C7, Pan02, and HEK239T were cultured in Dulbecco's Modified Eagle Medium (DMEM)(Gibco, USA) supplemented, and CFPAC1 in IMDM. All cell culture media were supplemented with 10% fetal bovine serum (FBS) (Gibco, USA) and 1% antibiotics. All cells were maintained at 37 °C in a humidified incubator with a 5% CO2. All cells were routinely authenticated through short tandem repeat (STR) genotyping and were tested negative for mycoplasma at regular intervals.

### Patient Samples

Sixty matched pairs of pancreatic cancer tissues and their corresponding adjacent normal tissues were collected from patients at Harbin Medical University Cancer Hospital for immunohistochemistry (IHC). Immunohistochemistry was performed on 116 PC tumor tissue sections for patient survival analysis, with 16 fresh tissue samples used for TRIM21 or EPHX1 detection, and RNA was extracted from 25 fresh tissue samples. All patients provided written informed consent, and the study was approved by the Ethics Committee of Harbin Medical University Cancer Hospital. The ethics approval number is KY‐2022‐38.

### Western Blot

Cellular proteins were separated by SDS‐PAGE on gradient gels ranging from 7.5% to 12.5% and transferred to 0.2 µm PVDF membranes. After blocking, the membranes were incubated with primary antibodies overnight at 4 °C. Following washes, membranes were treated with HRP‐conjugated secondary antibodies and visualized using enhanced chemiluminescence detection reagent.^[^ ^39^ ^]^ Antibodies used in experiments were presented in Table S6, Supporting Information.

### Co‐Immunoprecipitation

Sufficiently transfected cells were lysed in lysis buffer containing protease inhibitors, followed by sonication on ice. Take 60 µL of the supernatant after centrifugation, mix with loading buffer, and boil at 100 °C for 10 min. to generate input group. The remaining supernatant was incubated overnight at 4 °C with the designated Protein A/G magnetic beads (Anti‐His Magnetic Beads, Beyotime; Anti‐Flag Magnetic Beads, Selleck). The beads were then washed three times with pre‐chilled TBST, resuspended in buffer, and boiled for 10 min. The beads were removed using magnetic separation, followed by Western blot analysis. Prior to detecting ubiquitination in the Co‐IP experiment, cells were treated with 10 µM MG132 for 4 h before collecting.

### Immunofluorescence Colocalization Analysis

Cells cultured on glass slides fixed with Methanol at room temperature for 10 min. Following fixation, the cells were permeabilized using 0.3% Triton X‐100 for 15 min, followed by blocking with 5% bovine serum albumin (V900933, Sigma, German) for 30 min. After blocking, (EPHX1 antibody (1:100; GTX109360, GeneTex) and FLAG‐tag (1:100; # 8146S, CST) was applied and incubated overnight at 4 °C. The slides were rinsed with PBS three times and subsequently treated with secondary antibodies: Alexa Fluor 594 conjugate (Cat# ab150080, Abcom) and Alexa Fluor 488 conjugate (Cat# ab150113, Abcom), along with DAPI, for 30 min in a humidified chamber at 37 °C. Images were captured using a ZEISS LSM 900 Airyscan 2 confocal microscope and analyzed with ImageJ (2.15.1).

### C11‐BODIPY and ROS Staining

PC cells were seeded in 6‐well cell culture plates. The cells were treated with the respective drugs, followed by staining with 5 µmol L^−1^ C11‐bodipy (GC40165, GlpBio, USA) at 37 °C cell incubator for 30 min. After washing with PBS three times or more, were trypsinized and subsequently analyzed by flow cytometry. Similarly, after seeding the cells, they were treated with the 100 um mL^−1^ arachidonic acid (SA9940, Solarbio)72 h. The cells were incubated for 30 min in serum‐free culture medium containing 10 µmol L^−1^ DCFH‐DA, then digested with trypsin and analyzed using flow cytometry. Fluorescence intensity was measured with a flow cytometer.

For ROS fluorescence microscopy imaging, cells grown on glass slides, after undergoing the same arachidonic acid treatment as described above, the cells were incubated with DAPI for 30 min and then examined using an inverted microscope (Olympus BX53).

### Quantification of Arachidonic Acid

Stably transfected cells were cultured in a 5% CO₂ environment at 37 °C for 6 h in the absence of FBS. Cells were then collected by centrifugation for further analysis. ELISA assays were performed following the manufacturer's instructions with the ELISA kit (CB10700‐Hu, Coibo Bio, Shanghai). Absorbance was read at 450 nm using a microplate reader with full‐wavelength capability (TECAN Spark).

### Molecule Drug Screening

The PDB structure of TRIM21(P19474) was retrieved from the AlphaFold Protein Structure Database (https://alphafold.ebi.ac.uk/). The SDF chemical structures of C10 class (Lipid modifying agents) drugs were obtained from the DrugBank database. The SDF chemical structures of small‐molecule drugs classified under ATC Code level 1 in the DrugBank database were collected to form a compound library for screening. The 3D docking region on the target receptor protein TRIM21 was determined using the GetBox plugin in the PyMOL software (Schrödinger, New York, USA). Molecular docking of each small molecule in the compound library with the target protein docking region was conducted out with the AutoDock Vina software (version 1.1.2). The docking poses of small‐molecule drugs with the target protein were visualized and analyzed using PyMOL software.

### PDX Model and Therapeutic Experiments in Vivo

Fresh pancreatic cancer tumor samples were collected immediately after surgical excision and transported in chilled complete culture medium. The tissue was then sectioned into 2–3 mm fragments and implanted into NYG mice from Liaoning Changsheng biotechnology (Shenyang, China). Tumor growth in the xenografts was monitored twice weekly using calipers, and when the tumor volume reached about 0.1 cm^3^, passaging was initiated. Upon reaching the third passage, the drug administration experiment was formally initiated. The treatment group received the following: Gemcitabine was given intraperitoneally at a dose of 50 mg kg^−1^ weekly, while another group was given an equivalent volume of saline via intraperitoneal injection. Tumor growth was monitored every 3 to 4 days, and tumor volume was calculated using the formula V = length × width^2^. The tumor growth inhibition index (TGI) was calculated as [1 – (mean relative tumor volume of treated tumors)/ (mean relative tumor volume of control tumors)] × 100%. Statistical analysis of tumor growth was performed using GraphPad Prism v. 9.5.0. The ethics approval number is KY‐2022‐38.

### Statistical Analysis

Continuous variables were analyzed using the Student's *t*‐test for paired samples. To analyze the relationship between gene expression levels in the signature and the expression of TRIM21 and EPHX1, both Pearson and Spearman correlation analyses were used. Kaplan–Meier survival curves along with log‐rank tests were used to evaluate overall survival (OS) rates and curves for patients with PC. The results are expressed as means ± SEM or ± SD from three independent experiments, each conducted in duplicate. Statistical analyses were performed using GraphPad Prism v. 9.5.0 and R (version 4.3.1, http://www.bioconductor.org). In all experiments, a *p* value of less than 0.05 was deemed statistically significant. The results were indicated in the figures as follows: ns for *p* > 0.05; * for *p* < 0.05; ** for 0.001 ≤ *p* < 0.01; and *** for 0.0001 ≤ *p* < 0.001, **** for *p*< 0.0001.

### Ethics Statement

The studies involving humans were approved by the Ethics Committee of Harbin Medical University Cancer Hospital. The ethics approval number is KY‐2022‐38. Informed consents were obtained from all patients before surgery and participation of this study. All animal experiments were conducted in accordance with the approved protocol and other relevant guidelines and regulations. The raw data on tumor growth are available upon request from the corresponding author.

Additional details of the materials and methods can be found in Supplementary methods.

## Conflict of Interest

The authors declare no conflict of interest.

## Author Contributions

X.F., Y.D., and C.M. contributed equally to this work. Z. W. Li, C. J. Lou, C. Liu designed and conceived the study; Z. W. Li, C. Liu supervised the study, acquired funding; X.N. Fan, Y.S. Dai, C. F. Mo, H. Z. Li, X. D. Luan, G. T. Jiao, Z. Chen, Y. Y. Liao, L. Qu performed experiments and analyzed data; B. J. Wang, J. N. Yang, X. L. Lu, H. K. Yang provided advice and technical assistance; and X.N. Fan, Y.S. Dai, C. F. Mo, wrote the manuscript.

## Supporting information



Supporting Information

Supporting Information

Supporting Information

## Data Availability

The data that support the findings of this study are available from the corresponding author upon reasonable request.
