# Supplementary material for: TRIM21 Promotes Tumor Growth and Gemcitabine Resistance in Pancreatic Cancer by Inhibiting EPHX1‐Mediated Arachidonic Acid Metabolism
Source: Adv Sci (Weinh). 2024 Dec 30;12(8):2413674. doi: 10.1002/advs.202413674 (PMC11848624; doi:10.1002/advs.202413674)
Supplement: Supplementary file 1 — Supporting Information [file ADVS-12-2413674-s001.docx]

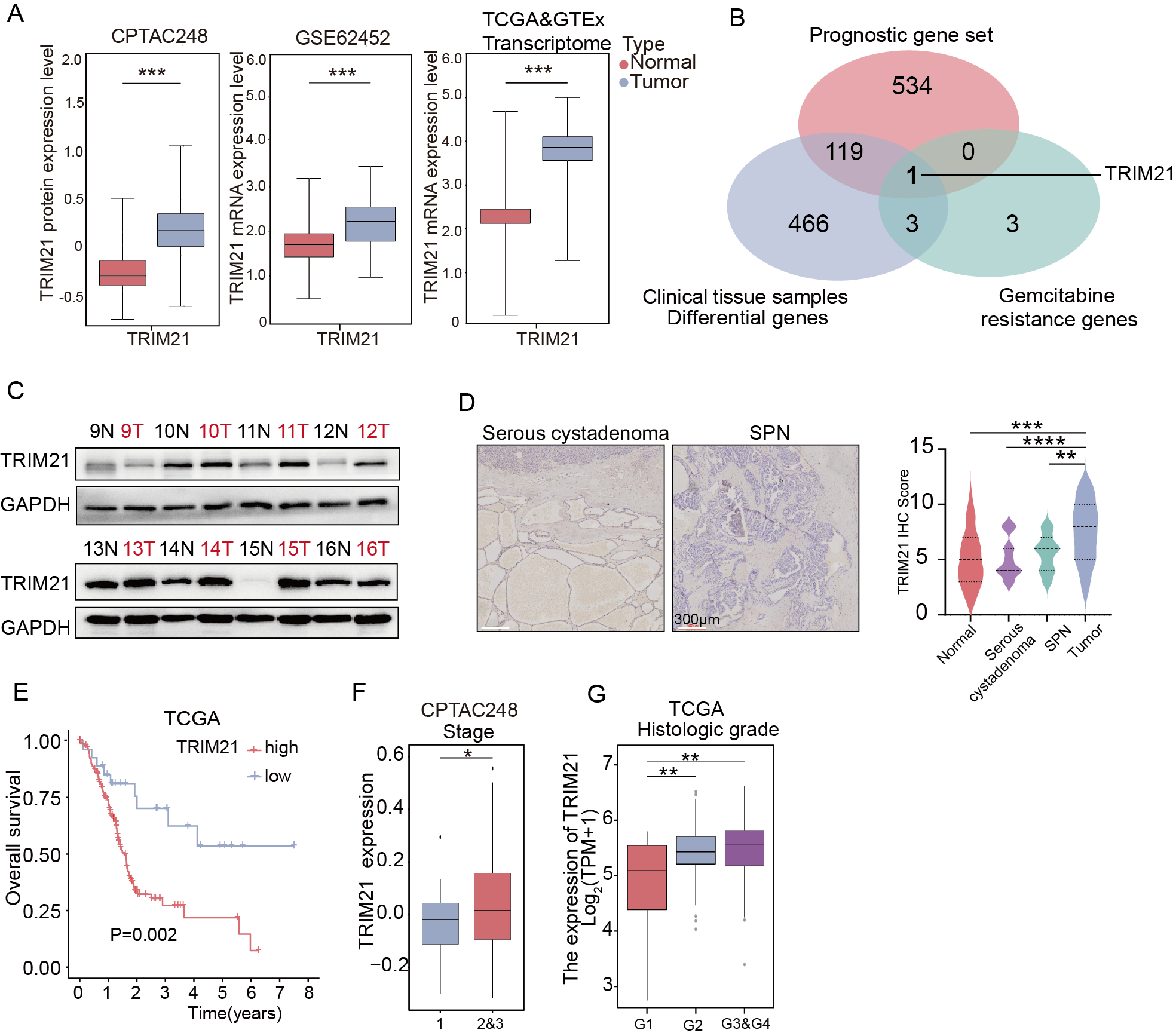


**Figure S1. TRIM21 is upregulated in pancreatic cancer and is associated with poor response to gemcitabine treatment. A.** TRIM21 levels in PC and adjacent non-cancerous from public databases CPTAC248, GSE62452, TCGA & GTEx. **B.** Venn diagram illustrating the overlapping genes associated with the prognostic gene set (CPTAC248 and TCGA databases), clinical tissue sample differential genes (CPTAC248, GSE62452, TCGA & GTEx), and gemcitabine-resistance genes from GEO (GSE 80617, GSE152121, GSE140077). **C.** Western blot analysis showing TRIM21 expression in the remaining pairs of fresh PC and adjacent non-cancerous tissues. **D.** IHC analysis was performed to examine the expression of TRIM21 in pancreatic serous cystadenomas and solid pseudopapillary neoplasm (SPN) (left). Scale bars, 300 µm. The expression levels and statistical analysis of TRIM21 were statistically analyzed in normal tissue, pancreatic serous cystadenomas, SPN, and pancreatic tumors(right). **E.** Kaplan-Meier survival analysis of PC patients in the TCGA database, correlating TRIM21 expression with survival probability. Statistical analysis was performed using the log-rank test: P = 0.002. **F.** Correlation between TRIM21 expression and PC stage in the CPTAC248 cohort, showing higher TRIM21 expression in stage II & III compared to stage I. **G.** Correlation between TRIM21 expression and histologic grade in the TCGA database, indicating increased TRIM21 expression with higher histologic grade. Statistical significance in the figures is indicated as follows: ns>0.05; * for P < 0.05; ** for 0.001 ≤ P < 0.01; *** for 0.0001 ≤ P < 0.001.


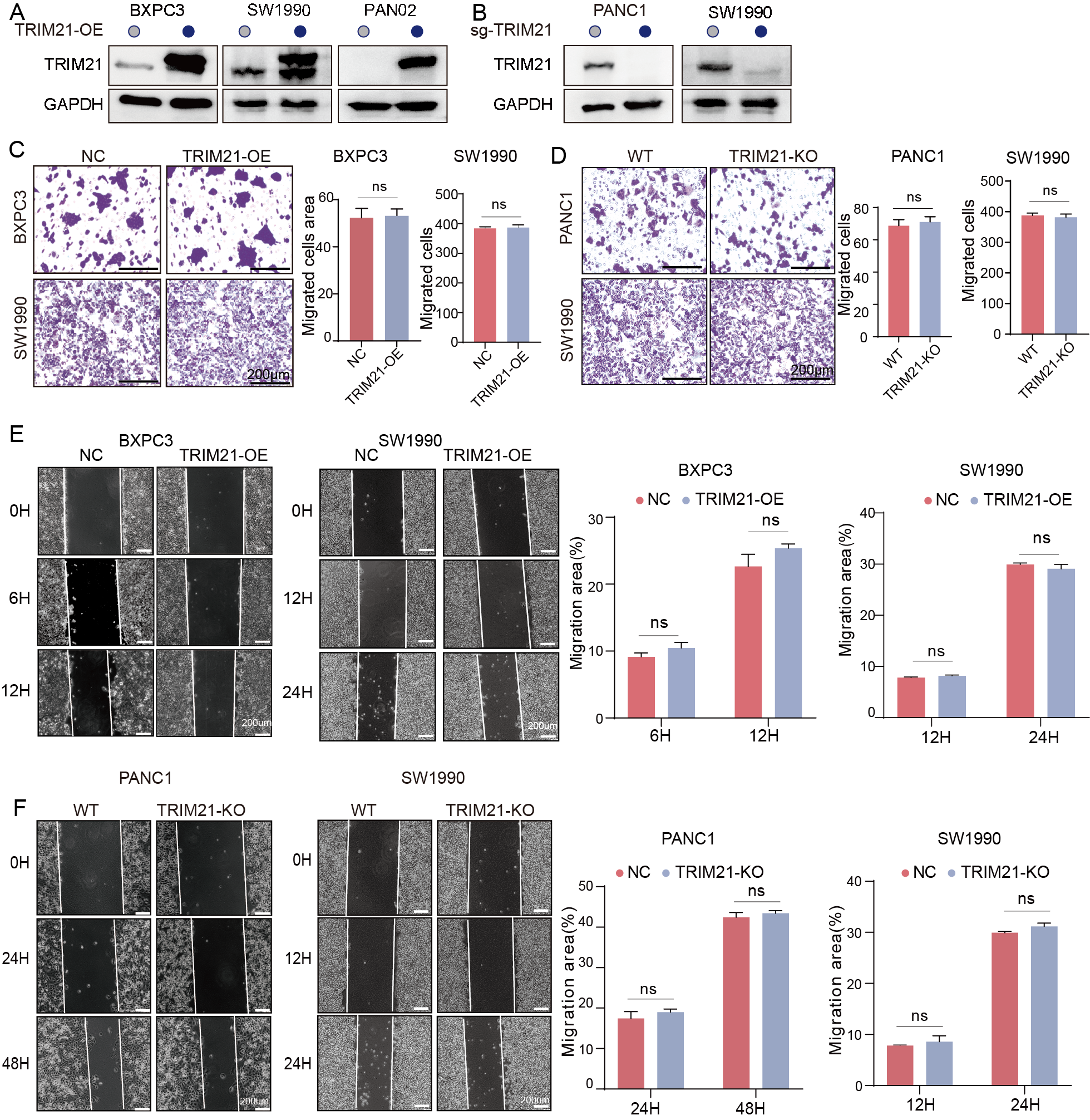


**Figure S2.** **TRIM21 promotes the proliferation of pancreatic cancer in vivo and vitro. A.B.** Overexpression infection efficiency in BXPC3, SW1990, and PAN02 cell lines, and TRIM21 knockout validation in SW1990 and PANC1 cell lines. **C.D.** Assess the migration capability after TRIM21 overexpression and TRIM21 knockout using the transwell assay. Scale bars, 200 µm. **E.F.** wound healing assay was used to assess the migration capability after TRIM21 overexpression and TRIM21 knockout. Scale bars, 200 µm, Statistical analysis for **C. D. E. F** was performed using the two-tailed unpaired t-test. Statistical significance in the figures is indicated as follows: ns>0.05;


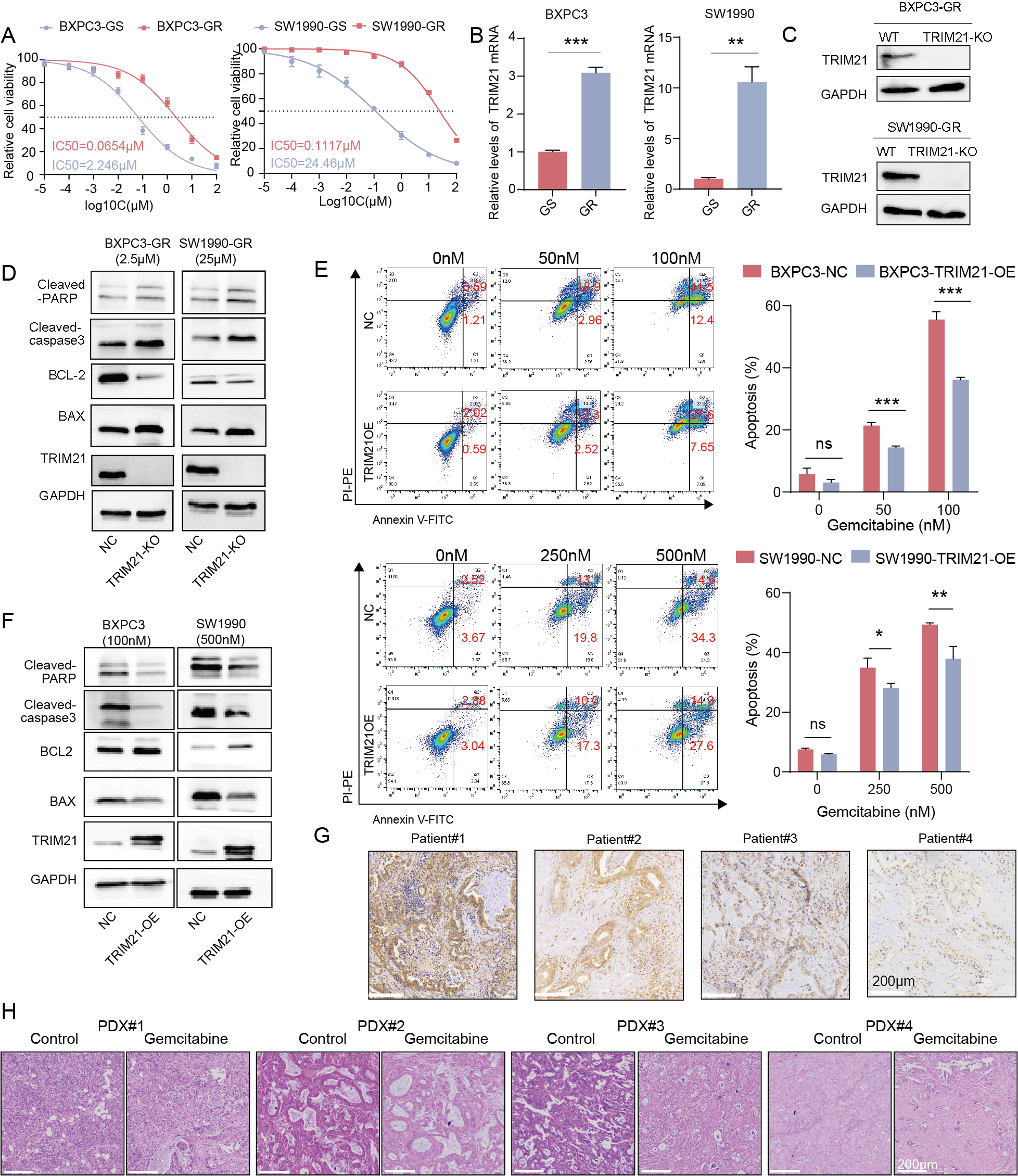


**Figure S3. TRIM21 enhances gemcitabine resistance in pancreatic cancer**

1. The IC50 values of gemcitabine were measured for both gemcitabine-sensitive (BXPC3-GS, SW1990-GS) and gemcitabine-resistant cell lines (BXPC3-GR, SW1990-GR). **B.** RT-PCR was conducted to evaluate the mRNA levels of TRIM21 in BXPC3-GS, BXPC3-GR, SW1990-GS, and SW1990-GR. **C.** TRIM21 was knocked out in BXPC3-GR and SW1990-GR, and the knockout efficiency was verified by Western blot analysis. **D.** BXPC3-GR and its TRIM21-knockout cells and SW1990-GR and its TRIM21-knockout cells were treated with gemcitabine, followed by the assessment of apoptosis-related proteins. **E.** Annexin-V/PI analysis was performed on NC and TRIM21-OE BXPC3 (upper) and SW1990 (lower) following 72h gemcitabine treatment. **F.** BXPC3 and its TRIM21-OE cells and SW1990 and its TRIM21-OE cells were treated with gemcitabine, followed by the assessment of apoptosis-related proteins. **G.** IHC shows the basal TRIM21 expression in the patients used for constructing PDX models. Scale bars, 200 µm. **H.** Hematoxylin and eosin (HE) staining was performed on the tissues of PDX mice between control and gemcitabine treatment group. Scale bars, 200 µm. Statistical analysis was performed using a two-tailed unpaired t-test. Statistical significance in the figures is denoted as follows: ** for 0.001 ≤ P < 0.01; *** for 0.0001 ≤ P < 0.001.


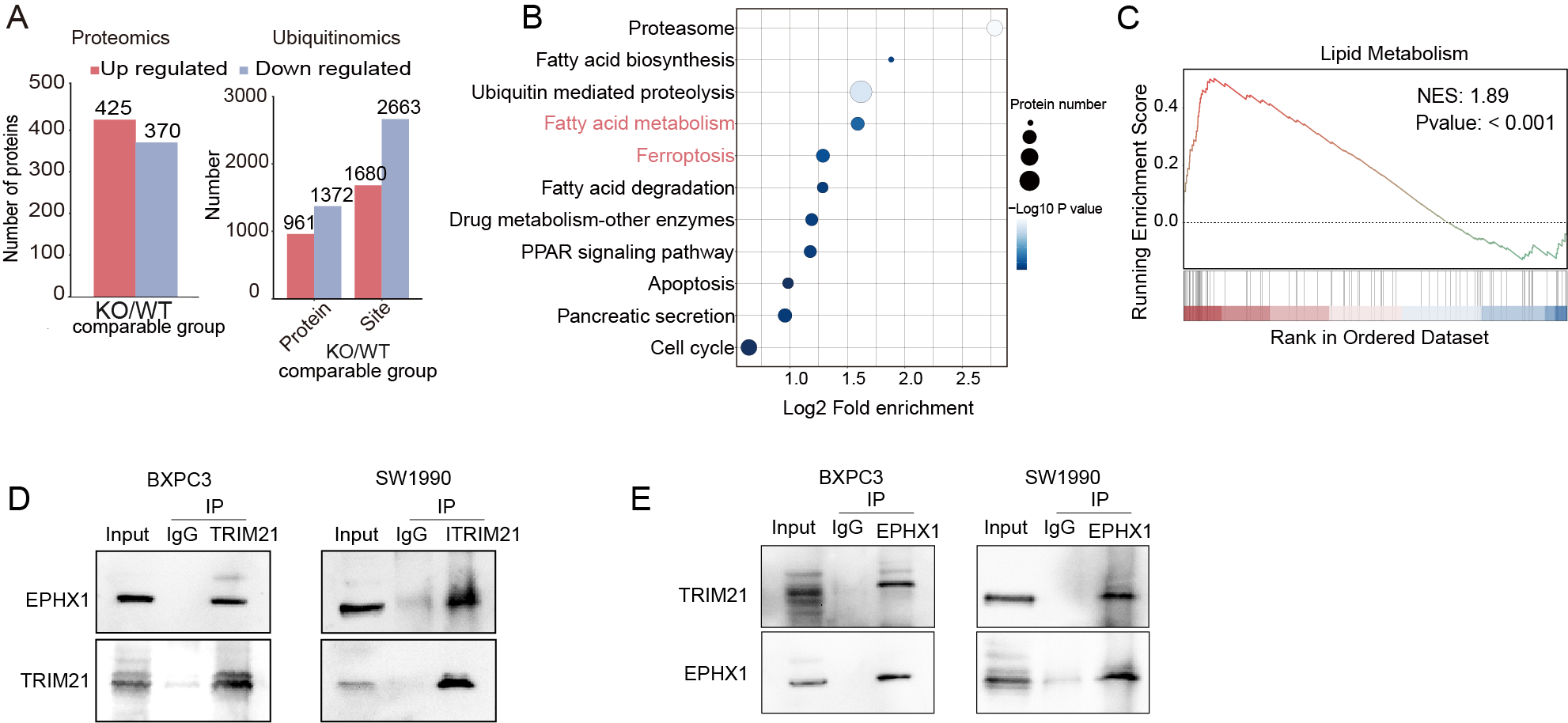


**Figure S4. TRIM21 promotes tumors through interacting with and enhancing degradation EPHX1. A.** The number of differentially expressed proteins in the PANC1-TRIM21-KO group compared to the PANC1-WT group in proteomics analysis (left), and the number of proteins and ubiquitination sites with differential ubiquitin modifications in the ubiquitin proteomics analysis (right). **B.** KEGG pathway analysis (P < 0.05) was performed on the differentially expressed proteins identified in the proteomic and ubiquitinomic analyses between PANC1-TRIM21-KO and PANC1-WT cells. **C.** GSEA shows that the differentially expressed proteins in the proteomic analysis are upregulated with lipid metabolism gene expression. **D.** Endogenous EPHX1 protein was immunoprecipitated using a TRIM21 antibody through the Co-IP. E. Endogenous TRIM21 protein was immunoprecipitated using a EPHX1 antibody through the Co-IP.


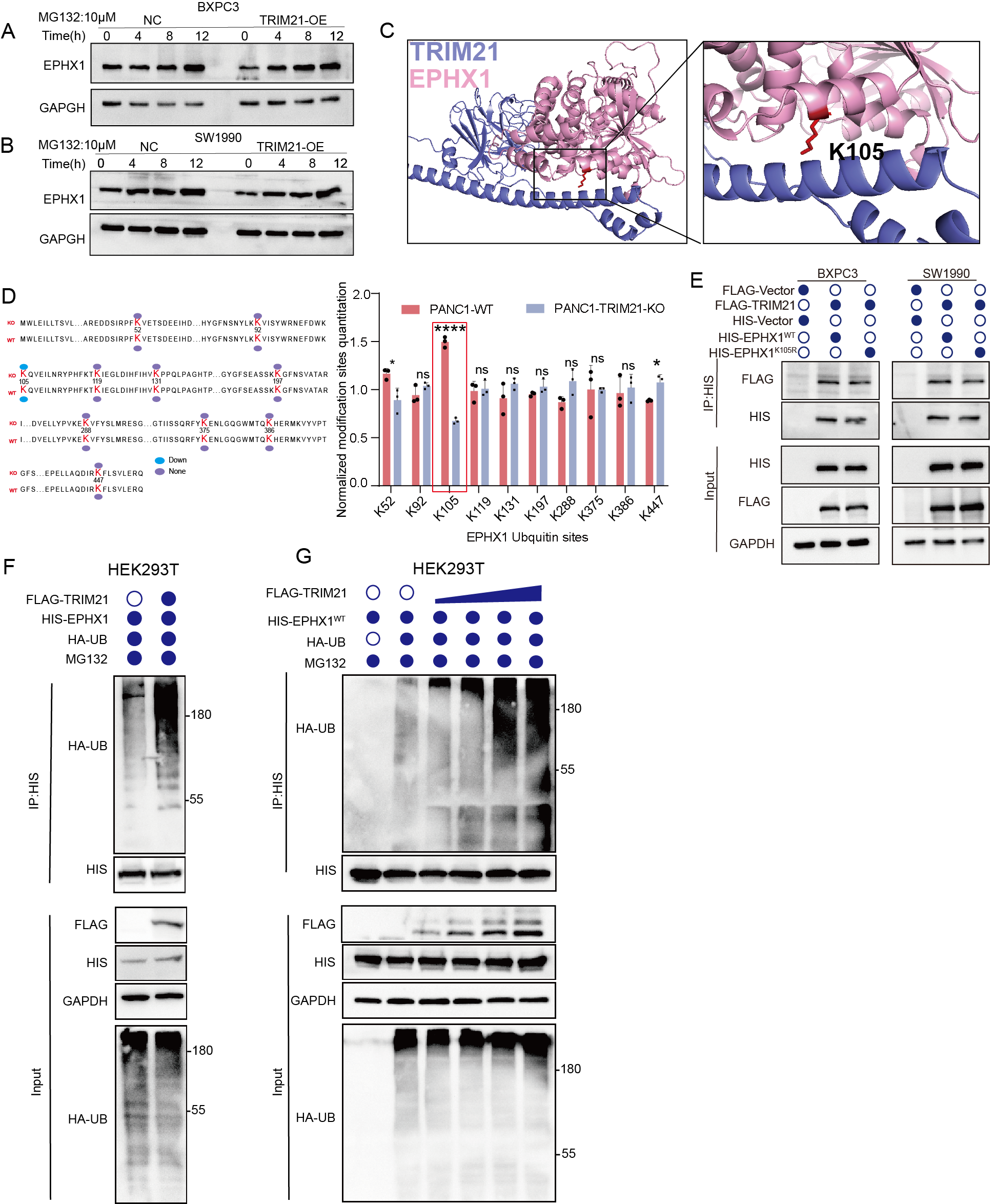


**Figure S5. TRIM21 via promoting K33-and K48-linked poly-ubiquitinates EPHX1 at lysine 105. A.B.** BXPC3 and SW1990 cells were treated with MG132 (10μM) following overexpression of FLAG-TRIM21 or in the NC group, and EPHX1 expression was assessed. **C.** The AlphaFold2 approach was used to visualize the interaction between TRIM21 and EPHX1 (left), with K105 representing the ubiquitination site of EPHX1. **D.** Ubiquitination sites of EPHX1 identified by ubiquitin proteomics (left) and quantitative changes in ubiquitination levels in the PANC1-TRIM21-KO group compared to the PANC1-WT group (right). A two-tailed unpaired T-test was applied, with differential modification defined as having a p-value <0.05 and |fold change| >1.5. **E.** Co-IP and Western blotting were performed in BXPC3 and SW1990 cells transfected with Flag-TRIM21 and either HIS-EPHX1 or HIS-EPHX1^K105R^. **F.** Co-IP followed by western blotting was used to detect the polyubiquitination levels of EPHX1 in HEK293T cells transfected with HIS-EPHX1, FLAG-TRIM21, and HA-UB. **G.** Co-IP followed by western blotting was used to detect ubiquitization level of EPHX1 after transfecting HIS-EPHX1, HA-UB and the increasing amounts of FLAG-TRIM21. Statistical analysis was performed using a two-tailed unpaired t-test. ns > 0.05; * for P < 0.05; and **** for P < 0.0001.


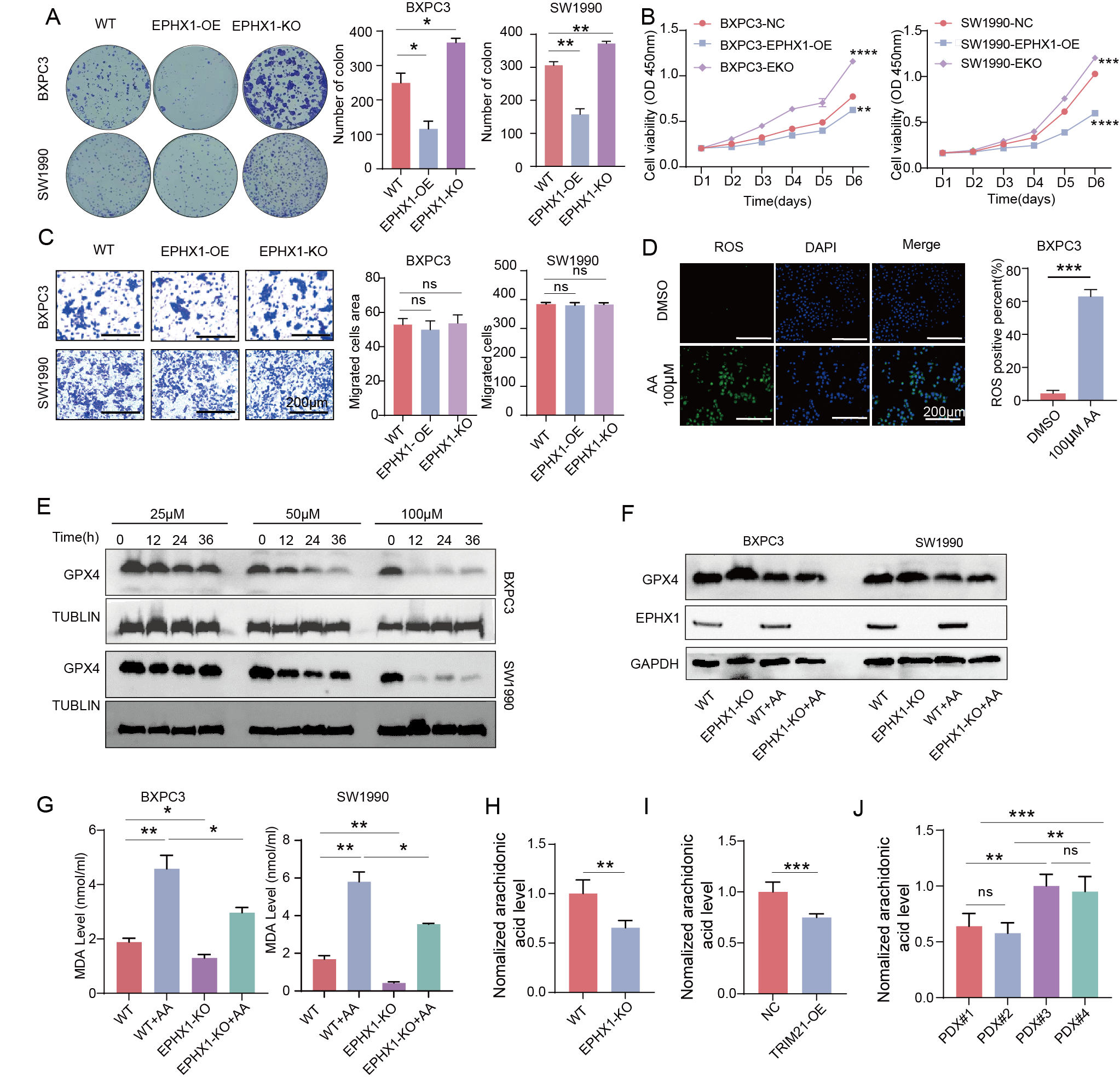


**Figure S6. EPHX1 mediates ferroptosis induced by arachidonic acid (AA) to inhibit PC proliferation**

**A.** Clonogenic assay in BXPC3 and SW1990 with EPHX1 overexpression and knockout. **B.** Cell proliferation assay in BXPC3 and SW1990 following EPHX1 overexpression and knockout. **C.** Transwell assay to assess migratory capacity in BXPC3 and SW1990 after EPHX1 overexpression and knockout. Scale bars, 200 µm. **D.** Immunofluorescence showing ROS levels in BXPC3 cells treated with AA (100 µM, 72 hours). Scale bars, 200 µm. **E.** Western blot analysis of GPX4 expression in BXPC3 and SW1990 cells treated with different concentrations of AA for specific durations. **F.** GPX4 expression in wild-type or EPHX1 knockout BXPC3 and SW1990 with or without AA (100 µM, 72 hours). **G.** Malondialdehyde (MDA) assay in wild-type or EPHX1 knockout BXPC3 and SW1990 with or without AA (100 µM, 72 hours). **H.I.J.K.** AA Normalized AA level in tumor of the subcutaneous xenograft model (SW1990-WT vs. EPHX1-KO) (**H**), the pancreatic cancer orthotopic model (PAN02-NC vs. TRIM21-OE) (**I**), and the control group of PDX models(**J**).Statistical analysis, Two-tailed unpaired T-tests were used for **A. C. D.** while two-way ANOVA was applied for **B**. Data are presented as mean ± SEM. Statistical significance is indicated as follows: ns > 0.05; * for P < 0.05; ** for 0.001 ≤ P < 0.01; *** for 0.0001 ≤ P < 0.001; and **** for P < 0.0001.


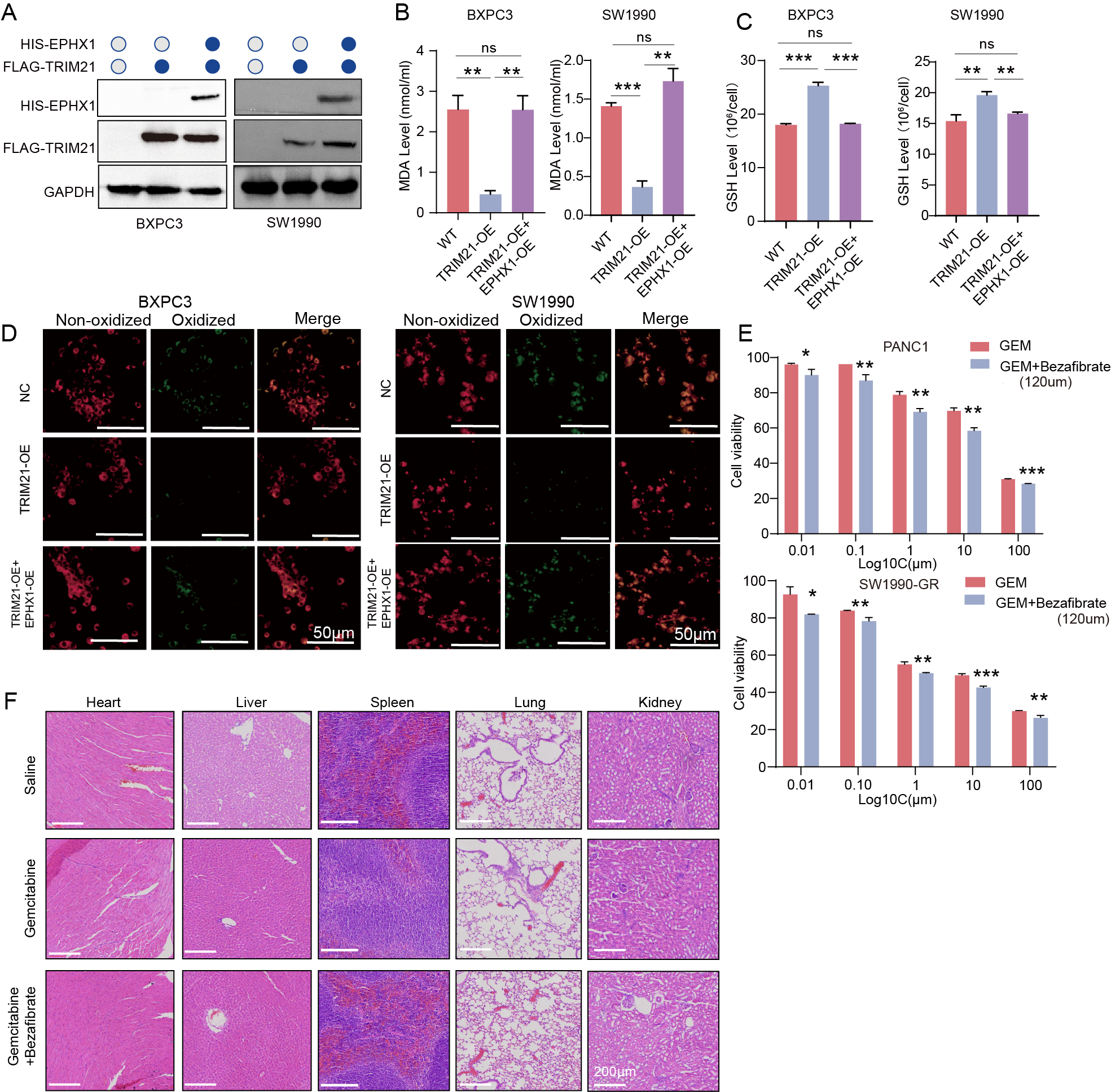


**Figure S7. Bezafibrate sensitizes gemcitabine by preventing the interaction between TRIM21 and EPHX1**

1. Western blot validation of FLAG-TRIM21 overexpression and co-overexpression of FLAG-TRIM21 and HIS-EPHX1. **B. C. D.** MDA assay(**B**), reduced GSH detection(**C**), C11 BODIPY 581/591-stained fluorescence microscope images(**D**) of WT group, TRIM21-OE group, and TRIM21 and EPHX1 co-overexpression group in BXPC3 and SW1990 cell lines. **E.** Cell viability assessment in PANC1 and SW1990-GR cells following treatment with gemcitabine alone or gemcitabine combined with Bezafibrate (120 µM, 72 hours). **F**. H&E staining of heart, liver, spleen, lung and kidneys across the different treatment groups in SW1990-GR subcutaneous xenograft tumor mice. Scale bars, 200 µm. Statistical analysis, Two-tailed unpaired T-tests were used for Statistical significance is indicated as follows: * for P < 0.05; ** for 0.001 ≤ P < 0.01; *** for 0.0001 ≤ P < 0.001.

**Supplemental Table 1. Clinical characteristics of the 60 paired PDAC patients.**

| **Category** | **N(%)(n=60）** |
| --- | --- |
| Gender  Male  Female | 41(68.3)  19(31.7) |
| Age  ≤60 years  ＞60 years | 35(58.3)  25(41.7) |
| Histological differentiation  Poorly  Moderately  Well | 12(20)  33(55)  15(25) |
| T stage  T1  T2  T3 | 10(16.7)  18(30)  32(53.3) |
| N stage  N0  N1+2 | 35(58.3)  25(41.7) |
| Pathologic stage  I  II  III  Perineural invasion  0  1 | 17(28.3)  19(31.7)  24(40)  49(81.7)  11(18.3) |

| **Category** | **TRIM21^low^**  **(%)(n=44）** | **TRIM21^high^**  **(%)(n=72）** |
| --- | --- | --- |
| Gender  Male  Female | 30(68.2)  14(31.8) | 48(66.7)  24(33.3) |
| Age  ≤60 years  ＞60 years | 31(70.5)  13(29.5) | 44(61.1)  28(38.9) |
| Histological differentiation  Poorly  Moderately  Well | 3(6.8)  24(54.5)  17(38.7) | 16(22.2)  42(58.3)  14(19.5) |
| T stage  T1  T2  T3 | 7(15.9)  14(31.8)  23(52.3) | 11(15.3)  17(23.6)  44(61.1) |
| N stage  N0  N1+N2 | 25(56.8)  19(43.2) | 51(70.8)  21(29.2) |
| Pathologic stage  I  II  III  Perineural invasion  0  1 | 11(25)  14(31.8)  19(43.2)  35(79.5)  9(20.5) | 21(29.2)  31(43)  20(27.8)  56(77.8)  16(22.2) |

**Supplemental Table 2. Clinical characteristics of the 116 PDAC patients**.

| **Category** | **Trim21^low^**  **(%)(n=22）** | **Trim21^high^**  **(%)(n=18）** |
| --- | --- | --- |
| Gender  Male  Female | 12(54.5)  10(45.5) | 11(61.1)  7(38.9) |
| Age  ≤60 years  ＞60 years | 14(63.6)  8(36.4) | 11(61.1)  7(38.9) |
| Therapeutic strategy  Gemcitabine  Gemcitabine+ Nab - Paclitaxel  Gemcitabine+ Oxaliplatin  Gemcitabine+Others | 1(4.6)  9(40.9)  5(22.7)  7(31.8) | 4(22.2)  6(33.3)  3(16.7)  5(27.8) |
| Response ratio  Response  No-response  Efficacy evaluation  PR  SD  PD | 17(77.3)  5(22.7)  8(36.4)  9(40.9)  5(22.7) | 7(38.9)  11(61.1)  2(11.1)  5(27.8)  11(61.1) |

**Supplemental Table 3. Clinical characteristics of the 40 PDAC patients with treatment**

**Supplemental Table 4.** **Clinical and pathological information of patients for PDX model.**

| **ID** | **Age** | | **Sex** | | **T**  **stage** | **N stage** | **Pathological**  **type** | | **Perineural invasion** | **Vascular invasion** |
| --- | --- | --- | --- | --- | --- | --- | --- | --- | --- | --- |
| Patient#1 | | 58 | | Male | 3 | 0 | Ductal adenocarcinoma | Positive | | Negative |
| Patient#2 | | 62 | | Female | 3 | 0 | Ductal adenocarcinoma | Positive | | Negative |
| Patient#3 | | 62 | | Male | 3 | 0 | Ductal adenocarcinoma | Positive | | Negative |
| Patient#4 | | 52 | | Female | 3 | 0 | Adenosquamous carcinoma | Positive | | Negative |

| **DrugBank ID** | **Affinity (kcal/mol)** | | **Residues** |
| --- | --- | --- | --- |
| DB08827 | -12.4 |  | |
| DB01599 | -11.6 | 371L | |
| DB00973 | -10.6 |  | |
| ­­­­DB13919 | -10.4 | 328Y | |
| DB01076 | -10.3 | 450F | |
| DB00808 | -10.2 |  | |
| DB00641 | -10 |  | |
| DB01095 | -10 | 395Q | |
| DB00227 | -9.9 |  | |
| DB08860 | -9.8 | 447S | |
| DB09279 | -9.5 |  | |
| DB01098 | -9.2 | 371L | |
| DB13422 | -9.2 |  | |
| DB01393 | -9 | 371L 395Q | |
| DB00177 | -8.9 |  | |
| DB00178 | -8.8 |  | |
| DB00439 | -8.8 | 450F | |
| DB01039 | -8.6 |  | |
| DB13433 | -8.6 | 196Q | |
| DB00175 | -8.5 | 395Q | |
| DB13873 | -8.4 |  | |
| DB09064 | -8.3 |  | |
| DB00790 | -8.2 |  | |
| DB00509 | -8.1 |  | |
| DB13460 | -8.1 |  | |
| DB13441 | -7.9 |  | |
| DB00381 | -7.7 |  | |
| DB08983 | -7.7 |  | |
| DB01241 | -7.6 |  | |
| DB11936 | -7.5 |  | |
| DB00722 | -7.3 |  | |
| DB13849 | -7 |  | |
| DB00636 | -6.5 |  | |
| DB00945 | -6 |  | |
| DB09055 | -5.4 | 297N | |
| DB00627 | -5.2 |  | |
| DB04377 | -5.1 | 297N | |
| DB13348 | -5.1 | 297N | |
| DB04145 | -4.8 |  | |

**Supplemental Table 5 Molecule drug screening for C10 class (Lipid modifying agents) of DrugBank database.**

| **Antibody** | **Supplier** | **Application** | **Cat** |
| --- | --- | --- | --- |
| TRIM21 | Abcam | IHC, WB | ab207728 |
| EPHX1 | GenTex | IHC, WB | GTX109360 |
| GAPDH | Proteintech | WB, IHC, IF/ICC, IP, ELISA | 10494-1-AP |
| Anti-β-Tubulin | Abmart | WB, IF, IHC, IP | M20005 |
| FLAG-tag | Cell Signaling Technology | WB, IP, IHC, IF, F | #8146 |
| HIS-tag | Cell Signaling Technology | WB, IP, IF, F, ChIP | #12698 |
| HA-tag | Cell Signaling Technology | WB, IP, IHC,IF,F ChIP | #3724 |
| Cleaved-PARP | Abmart | WB, IHC, IF | T55265 |
| Cleaved-Caspase 3 | Abmart | WB, IHC, IF/ICC, ELISA | [TA7022](http://www.ab-mart.com.cn/page.aspx?node=%2077%20&id=%2021621) |
| Bax | Abmart | WB, IHC, IP, FC | T40051 |
| Bcl-2 | Abmart | WB, IHC, IF, IFC | T40056 |
| GPX4 | Abmart | WB, IHC, ICC/IF | T56959 |
| Mouse Anti-Rabbit lgG HRP | Abmart | WB | M21006 |
| Goat Anti-Rabbit IgG-HRP | Abmart | WB | M21002 |
| KI-67 | Abcam | IHC-P, ICC/IF | ab15580 |
| PCNA | Affinity | WB, IHC, IF/ICC | AF0239 |

**Supplemental Table 6.** **Antibodies used in experiments**

**Supplemental Table 7**. **Primers for sgRNA.**

| **Application** | **Gene** |  | **Sequences** | |
| --- | --- | --- | --- | --- |
| sgRNA | H-TRIM21-1 | | CACCGTCTGCAGGAGAAGCTCCAGGGTTT |  |
|  | H-TRIM21-2 | | CACCGGAGCCTGTGAGCATCGAGTGGTTT |  |
|  | H-TRIM21-3 | | CACCGTCATCTCAGAGCTAGATCGAGTTT |  |
|  | H-TRIM21-4 | | CACCGCACACTCCTGAGTTCTGGAGGTTT |  |
|  | H-EPHX1-1 | | CACCGCTCCTTGTAGAAGCGCTGGGGTTT |  |
|  | H-EPHX1-2 | | CACCGCAGCATCCGCCCTTTCAAGGGTTT |  |
|  | H-EPHX1-3 | | CACCGGAATTCCGTATTGGTCCAGGGTTT |  |
|  | H-EPHX1-4 | | CACCGTGTCCAGTAGAGCATGACGTGTTT |  |

**Supplemental Table 8. Primers for qRT-PCR**

| **Application** | **Gene** |  | **Sequences 5’-3’** |
| --- | --- | --- | --- |
| Quantitative | H-TRIM21 | Forward  Reverse | TCAGCAGCACGCTTGACAAT  GGCCACACTCGATGCTCAC |
|  | H-EPHX1 | Forward  Reverse | TCTCCTACTGGCGGAATGAAT  CAAGGGCTTCGGGGTATGG |
|  | H-β-ACTIN | Forward  Reverse | CATGTACGTTGCTATCCAGGC  CTCCTTAATGTCACGCACGAT |

**Supplemental methods**

**Antibodies**

Antibodies used in experiments were presented in Supplemental Table 6.

**Establishment of gemcitabine-resistant cell lines**

Our group established GEM-resistant BXPC3 and SW1990 cell lines using a gradient concentration culture screening method. The IC50 values for wild-type BXPC3 and SW1990 cells were used as the initial culture concentrations. As the cells became fully resistant to the drug concentration, the drug concentration was increased following the same method. After several months of continuous selection, BXPC3 and SW1990 cells were successfully developed into gemcitabine-resistant cell line.

**Plasmid Construction and CRISPR‒Cas9-Mediated Gene Editing**

Flag-TRIM21, HIS-EPHX1, and HA-Ub were generated by cloning human EPHX1 cDNA and TRIM21 cDNA and Ub -cDNA into the pLVSIN-CMV-PURO vector, respectively. PCR was employed to generate point mutations or truncated fragments of TRIM21, EPHX1, or HA-Ub using plasmids encoding full-length Flag-TRIM21, HIS-EPHX1 or HA-Ub as templates, respectively. To establish TRIM21 and EPHX1 knockout cell lines, specific single guide RNAs (sgRNAs) targeting either the human EPHX1 or TRIM21 gene were cloned into the pCDH-EF1-MCS-CMV-copGFP-T2A-Puro vector. Primers for sgRNA was shown in Supplemental Table 7. After 48 hours, sort the GFP-positive cells, which are the successfully transfected cells, using flow cytometry (BD FACSMelody). Cells were subsequently transfected with these sgRNA plasmids. Following transfection, single cell colonies were isolated by seeding the cells into 96-well plates. The resultant clones were validated through Sanger sequencing and further confirmed via immunoblot analysis.

**Transfection, and Lentivirus Production**

The VP004-CMV-MCS-3fIag-EF1-fLUC-T2A-PURO-TRIM21 virus was purchased from General Biosystems (Anhui, China). Remaining lentivirus production was carried out by cotransfecting (Lipofectamine 3000, Thermo Fisher, USA) HEK293T cells with the lentiviral plasmid along with the packaging and envelope plasmids (psPAX2 and pMD2.G). Six hours post-transfection, the culture medium was refreshed. 72 hours after transfection, viral supernatants were harvested, centrifuged at 1000 rpm for 5 minutes, and filtered through a 0.45 μm filter (Millipore, MA, USA). Target cells were subsequently infected with the viral supernatant in the presence of polybrene (TA003, General Biosystems) for 8 hours. Following infection, cells were selected with 1 μg/mL puromycin for a minimum of one week, starting 48 hours post-infection.

**Quantitative real-time PCR (qRT-PCR)**

Total RNA was isolated from tissues and cell lines using Trizol reagent, followed by reverse transcription utilizing Takara PrimeScriptTM RT Master Mix. The resulting cDNA was amplified by PCR with SYBR Green Master PCR Mix (Applied Biosystems). All PCR assays were performed SYBR Premix Ex-Taq II kit (Takara, Tokyo, Japan) on the Quant Studio 3 Real-Time PCR System (Applied Biosystems, CA, USA). The β-ACTIN gene served as an internal control for normalizing data across samples. The relative expression levels of each target gene, compared to its calibration standard, were calculated using the 2^- (Ct - Cc) method, where Ct and Cc denote the average threshold cycle values adjusted to β-ACTIN. Primers for qRT-PCR was shown in Supplemental Table 8.

**Bioinformatics analysis**

The ubiquitination gene set was established by screening the IUUCD database (<https://ngdc.cncb.ac.cn/>) and the Gene Ontology (GO) database (<https://www.geneontology.org/>). Clinical sample analysis was performed using the TCGA & GTE, GSE62425, CPTAC270 and PDC00248 datasets. The datasets used to screen for gemcitabine resistance in pancreatic cancer are GSE152121 and GSE140077. Transcriptome and proteomics profiles for pancreatic ductal adenocarcinoma (PDAC) were acquired from multiple sources: The Cancer Genome Atlas (TCGA) [<https://portal.gdc.cancer.gov>], the Genotype-Tissue Expression (GTEX) project [<http://www.gtexportal.org>], and the Gene Expression Omnibus (GEO) [[https://www.ncbi.nlm.nih.gov/geo]](https://www.ncbi.nlm.nih.gov/geo%5D.%E2%80%9D)

**Proteomics and Ubiquitinomics**

Proteomics: The cell pellet was resuspended in lysis buffer (8 M urea, 1% protease inhibitor, 50 μM PR-619) and lysed by sonication. The lysate was then centrifuged at 12,000 g for 10 minutes at 4°C, and the supernatant was collected for protein quantification. Equal amounts of protein were digested, with the volume adjusted using the lysis buffer to ensure consistency. Trichloroacetic acid (TCA) was added to a final concentration of 20%, followed by vortexing and precipitation at 4°C for 2 hours. The precipitate was pelleted by centrifugation at 4500 g for 5 minutes, and the supernatant was discarded. The pellet was washed 2-3 times with pre-cooled acetone. After air-drying, the pellet was resuspended in TEAB at a final concentration of 200 mM and sonicated to disperse the precipitate. Trypsin was added at a ratio of 1:50 (enzyme) for overnight digestion. Dithiothreitol (DTT) was then added to a final concentration of 5 mM and incubated at 56°C for 30 minutes for reduction. Subsequently, iodoacetamide (IAA) was added to a final concentration of 11 mM and incubated at room temperature in the dark for 15 minutes. The peptides were dissolved in mobile phase A of the liquid chromatography and separated using the NanoElute ultra-high-performance liquid chromatography (UHPLC) system. The separated peptides were ionized through a capillary ion source and analyzed using the timsTOF Pro mass spectrometer for data acquisition.

Prior to liquid chromatography-mass spectrometry (LC-MS) analysis, peptide modification enrichment was performed. The peptides were dissolved in IP buffer (100 mM NaCl, 1 mM EDTA, 50 mM Tris-HCl, 0.5% NP-40, pH 8.0) and the supernatant was transferred to pre-washed resin (PTM1104 PTM Bio). The mixture was incubated overnight on a shaker at 4°C. The resin was washed four times with IP buffer and twice with deionized water. The peptides bound to the resin were eluted with 0.1% trifluoroacetic acid, with three consecutive elutions performed. The eluent was collected and vacuum-dried. After drying, desalting was performed according to the C18 ZipTips protocol, followed by another round of vacuum drying before proceeding to LC-MS analysis.

**Quantitative Lipidomics Analysis**

After thawing on ice, add 100 μL of ultrapure water extract (containing protease inhibitors, PMSF, and EDTA) to resuspend the cell pellet. Separate 50 μL of the cell suspension and add 500 μL of a mixture (including methanol, MTBE, and an internal standard mixture). Vortex the mixture for 15 minutes, then add 100 μL of water, vortex for an additional 1 minute, and centrifuge at 12,000 rpm for 10 minutes at 4°C. Collect 200 μL of the supernatant and concentrate it. Dissolve the resulting powder in 200 μL of reconstitution solution, then store it at -80°C. Finally, transfer the solution to a sample vial for LC-MS/MS analysis. The remaining 50 μL of the cell suspension is subjected to three freeze-thaw cycles, centrifuged at 12,000 rpm for 10 minutes, and the supernatant is collected to measure protein concentration. The sample extract is analyzed using an LC-ESI-MS/MS system (UPLC, ExionLC AD, <https://sciex.com.cn/>; MS, QTRAP® 6500+ System, <https://sciex.com/>). LIT and triple quadrupole (QQQ) scans are performed on a triple quadrupole-linear ion trap mass spectrometer (QTRAP® 6500+ LC-MS/MS System) equipped with an ESI Turbo Ion-Spray interface, operating in both positive and negative ion modes, and controlled by Analyst 1.6.3 software (Sciex).

**Cell Proliferation and Migration Assays**

For the cell proliferation assay, cells were plated at a density of 500 cells per well in 96-well plates. Proliferation was assessed using the Cell Counting Kit-8 (CCK8) (CA1210, Solarbio).To evaluate cell migration, cells were seeded at a density of 3*10^5 cells per well in 24-well transwell plates. The lower chamber was filled with medium supplemented with 10% fetal bovine serum (FBS), while the upper chamber contained medium with 0% FBS. After 24 hours, non-migratory cells in the upper chamber were removed using a cotton swab. Migrating cells that adhered to the lower surface of the filter were fixed, stained with hematoxylin, and counted in three randomly selected fields from each chamber.

**Subcutaneous Tumor Transplantation Model in Mice and Treatment Studies**
Six-week-old female BALB/c-nude mice, sourced from Liaoning Changsheng biotechnology (Shenyang, China), were subcutaneously injected with 5 × 10⁶ cells into their right flanks. After 14 days tumors developed, treatment commenced with equivalent volume saline; gemcitabine administered in vivo (50 mg/kg on a weekly, intraperitoneally; and a combination of gemcitabine (50 mg/kg on a weekly, intraperitoneally) with bezafibrate (100 mg/kg daily, orally). Tumor dimensions were regularly measured every other day using Vernier calipers, and the volume was calculated using the formula: V = [length × (width)²]/2. Mice were euthanized if any tumor dimension exceeded 1.5 cm. All animal experiments were carried out with the approval of Harbin Medical University. Animal studies were reported incompliance with the ARRIVE guidelines.

**Orthotopic Pancreatic Cancer Mouse Model**

For the orthotopic model, C57BL/6 mice were injected with 5× 10^5 Pan02-pLV-control-luc or Pan02-pLV-TRIM21-luc cells in Matrigel (356234, Solarbio, Beijing) into the pancreatic tail. Utilizing the IVIS Spectrum imaging system (Caliper Life Sciences, USA), detection of orthotopic tumor commenced from the 14th day following injection.  Tumor growth was analyzed by bioluminescent imaging.

**Malondialdehyde (MDA) Assay**
The level of malondialdehyde (MDA) was assessed using an MDA assay kit (Solarbio, BC0025). Treated pancreatic cancer cells were harvested, washed, and lysed in extraction buffer. The samples were then centrifuged at 8000 g for 10 minutes at 4 °C. After centrifugation, 0.1 mL of the supernatant was mixed with 0.3 mL of the working solution and incubated at 100 °C for 60 minutes. After cooling to room temperature, the final supernatant was collected and centrifuged again at 10000 g for 10 minutes. The absorbance at 532 nm and 600 nm was measured using a microplate reader. MDA levels were calculated based on the manufacturer's instructions using the following formula:

MDA level in cell lysates (nmol)
=53.763×(A532−A600)

**GSH Detection**
The level of reduced glutathione (GSH) was measured using a GSH assay kit (Solarbio, BC1175). Treated pancreatic cancer cells were harvested, washed, and lysed in extraction buffer. The samples were then centrifuged at 12000 g for 10 minutes at 4 °C. After centrifugation, 0.02 mL of the supernatant was mixed with 0.18 mL of the working solution and incubated at room temperature for 2 minutes. The absorbance at 412 nm was then measured using a microplate reader. The GSH content is then calculated by a standard curve.
